# Comparative Plastid Genomics of Neotropical *Bulbophyllum* (Orchidaceae; Epidendroideae)

**DOI:** 10.3389/fpls.2020.00799

**Published:** 2020-07-03

**Authors:** Michelle Zavala-Páez, Leila do Nascimento Vieira, Valter Antônio de Baura, Eduardo Balsanelli, Emanuel Maltempi de Souza, Marco Cerna Cevallos, Mark W. Chase, Eric de Camargo Smidt

**Affiliations:** ^1^Departamento de Botânica, Universidade Federal do Paraná, Curitiba, Brazil; ^2^Departamento de Bioquímica e Biologia Molecular, Universidade Federal do Paraná, Curitiba, Brazil; ^3^Grupo de Investigación Nunkui Wakan, Universidad Politécnica Salesiana, Quito, Ecuador; ^4^Royal Botanic Gardens, Kew, Richmond, United Kingdom; ^5^Department of Environment and Agriculture, Curtin University, Perth, WA, Australia

**Keywords:** plastid genome, Neotropical orchids, molecular evolution, next-generation sequencing, molecular markers

## Abstract

Pantropical *Bulbophyllum*, with ∼2,200 species, is one of the largest genera in Orchidaceae. Although phylogenetics and taxonomy of the ∼60 American species in the genus are generally well understood, some species complexes need more study to clearly delimit their component species and provide information about their evolutionary history. Previous research has suggested that the plastid genome includes phylogenetic markers capable of providing resolution at low taxonomic levels, and thus it could be an effective tool if these divergent regions can be identified. In this study, we sequenced the complete plastid genome of eight *Bulbophyllum* species, representing five of six Neotropical taxonomic sections. All plastomes conserve the typical quadripartite structure, and, although the general structure of plastid genomes is conserved, differences in *ndh*-gene composition and total length were detected. Total length was determined by contraction and expansion of the small single-copy region, a result of an independent loss of the seven *ndh* genes. Selection analyses indicated that protein-coding genes were generally well conserved, but in four genes, we identified 95 putative sites under positive selection. Furthermore, a total of 54 polymorphic simple sequence repeats were identified, for which we developed amplification primers. In addition, we propose 10 regions with potential to improve phylogenetic analyses of Neotropical *Bulbophyllum* species.

## Introduction

*Bulbophyllum* Thouars is the largest Pantropical genus of Orchidaceae, with ∼2,200 species (WCSP, 2018). However, its distribution is not homogeneous over the entire range. With hundreds of species, the Paleotropics are by far the richest, followed by Africa and the Neotropics, the last with just ∼60 species (Smidt et al., 2011; Pridgeon et al., 2014). In the Neotropics, the genus is represented by six clades, two north of the Equator and four mainly in the Brazilian Atlantic Rainforest and Cerrado (Smidt and Borba, 2007; Smidt et al., 2011).

*Bulbophyllum* species are recognized worldwide not only for their diversity but also for the potential use of their compounds in the fields of traditional medicine and agricultural pest management. These orchids contain aromatic compounds that have properties of use in human health, such as in treatment of tumors and conception problems and production of antifebriles (Wu et al., 2006; Chen et al., 2008; Lalitharani et al., 2011). Furthermore, the volatile compounds of *Bulbophyllum* species are used in integrated pest management strategies. Tephritid flies, pests of many crops, can be attracted to traps using floral fragrances of *Bulbophyllum* (Tan et al., 2006; Tan and Nishida, 2007; Jaleel et al., 2018).

Owing to their species diversity and high levels of endemism, *Bulbophyllum* species have been widely studied in the Neotropics (Smidt and Borba, 2007; Smidt et al., 2007; Mancinelli and Smidt, 2012). As a result, multiple taxonomic (Borba et al., 1998; Ribeiro et al., 2008; Nunes et al., 2014, 2015, 2017) and phylogenic (Smidt et al., 2011, 2013) studies have been published. In molecular phylogenetic studies of Neotropical *Bulbophyllum* species, plastid [*psbA-trnH* and *trnS-trnG* intergenic spacers (IGSs)] and nuclear [internal transcribed spacer (ITS) spacer] markers have been employed (Smidt et al., 2011, 2013). However, despite these studies, some species complexes need further investigation to aid in species delimitation and provide evidence of natural hybridization/introgression (Borba and Semir, 1998; Azevedo et al., 2006; Mancinelli and Smidt, 2012).

Previous research (Zhitao et al., 2017) has demonstrated that the plastid genome can be an effective tool in population genetics, but ancestral polymorphisms can be problematic in some taxa (Dodsworth et al., 2020). In addition, the study of the plastid genome for phylogeographical analyses has great potential (Mariac et al., 2014; Smidt et al., 2020). In Orchidaceae, with the exception of several mycoheterotrophic species (Feng et al., 2016; Yuan et al., 2018), the plastid genome has the typical angiosperm quadripartite structure: two inverted repeated (IR) regions separated by a small (SSC) and large single-copy (LSC) regions (Chang et al., 2006; Delannoy et al., 2011; Pan et al., 2012; Yang et al., 2013; Luo et al., 2014; Zhitao et al., 2017; Smidt et al., 2020). The size of the plastid genome within orchids is relatively small, ranging from 0.01 to 0.17 Mb (Lin et al., 2015; Schelkunov et al., 2015). Gene content and order are generally conserved, but some variations have been reported in length, composition of the *ndh-*gene complex, and loss of many photosynthesis-related genes in mycoheterotrophic species (Graham et al., 2017; Zhitao et al., 2017).

In this article, we present complete plastid genomes for eight *Bulbophyllum* species, representing five of six Neotropical taxonomic sections. First, we compared gene content and order and codon use. Second, codons under positive selection in protein-coding genes are identified. Third, we determined locations of long- and short repeated sequences in these genomes, including those with potential as microsatellite markers. Fourth, sequence variability (SV) is calculated to determine the 10 most-variable regions, for which we provide PCR primer sequences. Finally, performance of the most-variable regions in these *Bulbophyllum* species is evaluated using a phylogenetic analysis. Our results provide information for species identification and increase phylogenetic resolution among *Bulbophyllum* species. Also, more effective molecular markers can produce results with implications for species management and conservation.

## Materials and Methods

### Plastid Genome Sequencing and Assembly

Plastid-enriched DNA (ptDNA) was extracted from fresh leaves of eight *Bulbophyllum* species (Table 1) using the Sakaguchi et al. (2017) methodology, reducing the amount of material required for plastid isolation. Afterward, ptDNA was extracted following the Doyle and Doyle (1987) protocol. Purification was performed with DNA Clean and Concentrator kit (Zymo Research, Orange, CA, United States).

**TABLE 1:** *Bulbophyllum* species sequenced, taxonomy, and collection data.

Species	Section	Locality	Collector/collection number/herbarium
*Bulbophyllum weddellii* (Lindl.) Rchb.f	*Didactyle*	Brazil, Minas Gerais, Serra da Piedade	*Fiorini 21* (HBCB)
*Bulbophyllum exaltatum* Lindl.	*Didactyle*	Brazil, Minas Gerais, Santa Rita de Caldas	*Fiorini 218* (HBCB)
*Bulbophyllum steyermarkii* Foldats	*Furvescens*	Ecuador, Azuay, Gualaceo	*Cerna 4403* (UPS)
*Bulbophyllum epiphytum* Barb.Robr.	*Micranthae*	Brazil, Paraná, Pirai do Sul	*Smidt 1084* (UPCB)
*Bulbophyllum mentosum* Barb.Robr.	*Micranthae*	Brazil, Minas Gerais, Parque Nacional Sempre Vivas	*Fiorini 323* (HBCB)
*Bulbophyllum regnellii* Rchb.f.	*Napelli*	Brazil, Paraná, Piraquara Mananciais da Serra—Reservatório Carvalho	*Smidt 1081* (UPCB)
*Bulbophyllum granulosum* Barb.Rodr	*Napelli*	Brazil, Santa Catarina, Pico Garuva	*Mancinelli 1059* (UPCB)
*Bulbophyllum plumosum* Barb.Rodr.	*Xiphizusa*	Brazil, Paraná, Tibagi—Parque Estadual do Guartelá	*Imig 606* (HAC)

DNA was sequenced on an Illumina MiSeq^®^ following instructions of DNA Nextera XT Sample Prep Kit (Illumina^TM^). The raw data were trimmed according to default parameters (i.e., quality limit = 0.05; ambiguous limit = 2; minimum read length = 30 bp) of CLC Genomics Workbench 8.0^1^, and a hybrid reference-guided *de novo* assembly approach was performed to assemble the genomes. First, the trimmed pair-end reads were mapped onto the complete plastid genome sequence of *Dendrobium officinale* Kimura & Migo (NC_024019.1) as a reference to generate a consensus sequence. The consensus sequence was extracted, excluding regions with low coverage (threshold < 8) and length fraction of 0.5. Afterward, the trimmed pair-end reads were *de novo* assembled using the following parameters: automatic word size, automatic bubble size, minimum contig length = 300, map reads back to contigs using mismatch cost = 2, insertion cost = 3, deletion cost = 3, length fraction = 0.5, and similarity fraction = 0.8. The multiple contigs created through the *de novo* assembly strategy were used in the gap-closing process, creating a consensus sequence developed with these high-quality contigs.

At this stage, there were six physical gaps in *Bulbophyllum exaltatum* Lindl. (3), *Bulbophyllum epiphytum* Barb.Robr (2), and *Bulbophyllum steyermarkii* Foldats (1). Three of them were resolved by amplification with primers specific to the flanking regions and Sanger sequencing on an ABI 3500 (Applied Biosystems). The others were located in AT-rich regions of repetitive sequences. These three gaps are small (9, 40, and 63 bp), and their length was estimated by reference mapping to a closely related *Bulbophyllum* species. Tentative errors sites were identified and manually checked by mapping the Illumina pair-end raw reads onto the assembled plastid genome. Assembled errors were characterized by mismatch readings or an abnormal read-mapping depth.

Dual Organellar GenoMe Annotator software (Wyman et al., 2004) and Geneious R7 (Kearse et al., 2012) were used to annotate the plastid genomes, using default values to predict genes coding for proteins (CDSs), transfer RNAs (tRNAs), and ribosomal DNAs (rDNAs). All genomes were checked manually, and the codon positions were determined by BLASTX against the National Center for Biotechnology Information (NCBI) protein database using the *D. officinale* plastid genome as a reference. Gene maps were drawn by Organellar Genome DRAW V1.1 (Lohse et al., 2007).

The newly assembled plastomes were deposited in GenBank (Table 2; accession numbers MN604056, MN737573, MN580547, MN604059, MN604054, MN604055, MN604057, and MN604058).

**TABLE 2:** General characteristics of the plastid genomes of eight *Bulbophyllum* species.

Species	*Bulbophyllum mentosum*	*Bulbophyllum epiphytum*	*Bulbophyllum plumosum*	*Bulbophyllum weddellii*	*Bulbophyllum exaltatum*	*Bulbophyllum granulosum*	*Bulbophyllum regnellii*	*Bulbophyllum steyermarkii*
GenBank accession	MN604056	MN737573	MN580547	MN604059	MN604054	MN604055	MN604057	MN604058
Total length (bp)	150,217	147,546	146,401	151,355	150,410	151,112	151,493	146,720
Length of LSC	83,642	82,354	83,260	83,450	83,335	84,492	84,868	83,488
Length of SSC	13,895	13,497	11,089	16,049	15,380	15,690	15,541	11,321
Length of IR	26,340	25,847	26,026	25,928	25,847	25,465	25,542	25,955
Total GC content (%)	36.7	36.7	36.6	36.6	36.8	36.7	36.7	36.7
LSC GC content (%)	34.1	34.1	33.7	34.7	34.2	34.3	34.2	34.2
SSC GC content (%)	28.3	28.5	27.6	28.9	29.1	29.1	29.1	27.9
IR GC content (%)	43.1	43.2	43	43.2	43.3	43.1	43.1	42.8
Number of genes	102	102	102	102	103	102	102	102
Number of CDS	68	68	68	68	69	68	68	68
Number of tRNA genes	30	30	30	30	30	30	30	30
Number of rRNA genes	4	4	4	4	4	4	4	4
Number of pseudogenes	10	9	7	10	8	10	10	6

*LSC, large single copy; SSC, small single copy; IR, inverted repeated region; tRNA, transfer RNA; rRNA, ribosomal RNA.*

### Plastid Genome Features

For each plastid genome, length, numbers of CDSs, tRNAs, and rDNAs were plotted. Genes in the inverted repeats and with two or more introns were identified. Junction positions between single-copy and IR regions were compared. Guanine-cytosine (GC) content was calculated using Geneious R7 (Kearse et al., 2012).

### Codon Usage

All CDSs for each plastid genome were extracted using Geneious R7 (Kearse et al., 2012) to determine the distribution of codon usage. The relative synonymous codon usage (RSCU) ratio was calculated using the R package SeqinR (Charif and Lobry, 2007). Relative synonymous codon usage values greater than 1 represent codons used more frequently than expected, and values less than 1 signify the opposite. RSCU distributions were illustrated in the form of heatmaps using Heatmapper (Babicki et al., 2016).

### Molecular Evolution of Protein-Coding Genes

A Bayesian inference (BI) approach was applied to identify amino acid sites in all CDSs under positive or purifying selection. The evolutionary model M8 was run on The Selecton server website (Stern et al., 2007). When positive selection was detected, a likelihood ratio test was run with the M8a (null) and M8 (alternative) models to test whether positive selection was significant. Furthermore, gene divergence analysis was performed with Selecton using the default parameters. Pairwise distances and branch lengths were computed using the maximum likelihood (ML) criterion under a codon model (Nielsen and Yang, 1998).

### Characterization of Repeat Sequences and Simple Sequence Repeats

Direct, reverse, and palindromic repeat sequences were identified by REPuter online program (Kurtz et al., 2001) according to a Hamming distance of 3.90% sequence identity and minimum repeat size of 30 bp. Simple sequence repeats (SSRs) were located using the MISA-web program (Beier et al., 2017) with search parameters set to the following: ≥10 repeat units for mononucleotide SSRs, ≥5 repeat units for dinucleotide SSRs, and ≥3 repeat units for dinucleotide, tetranucleotide, pentanucleotide, and hexanucleotide SSRS. Microsatellites primers were designed using Websat (Martins et al., 2009) for polymorphic SSRs present in at least four species. The following parameters were used: product length of 100–500 bp, primer length of 18–27 bp, and GC content of 40–60% with 1°C as the maximum difference between primer melting temperatures.

### Hypervariable Regions

To identify the 10 most-variable regions, all plastid genomes were aligned with the progressive Mauve algorithm (Darling et al., 2004). Then, the CDS, introns, and IGSs with minimum length of 150 bp flanked by the same region were manually extracted. The number of mutations and indel events was calculated by DnaSP v5 (Rozas et al., 2004), and their rate was calculated per 100 bp. Sequence variability was calculated as follows: (number of mutations + number of indel events)/(conserved sites + number of mutations + number of indel events) × 100% (Shaw et al., 2014). Due to indels having higher levels of homoplasy than mutations, indels were considered as events instead of sites in the alignment (Ingvarsson et al., 2003). Also, potentially parsimony informative sites were counted by DnaSP v5 program.

Specific primers were designed for the 10 most-variable regions using PRIMER3^2^ with the following parameters: maximum product length of 1,000 bp, primer length of 18–27 bp, and GC content of 40–60% with 2°C as the maximum difference between primer melting temperatures.

### Phylogenetic Analysis

To evaluate the performance of the marker regions identified in this study, phylogenetic analyses were performed for two datasets: (1) complete plastid genome sequences and (2) the 10 most-variable regions. Individual sequence alignments were performed using MAFFT and then combined for each dataset using SequenceMatrix (Vaidya et al., 2011).

Maximum parsimony (MP), BI, and ML analyses were run for each set. Maximum parsimony analyses were performed with PAUP v4b10 (Swofford, 2003), with a member of the sister genus of *Bulbophyllum*, *Dendrobium* [*Dendrobium aphyllum* (Roxb.) C.E.C.Fisch; NC_035322.1], as the outgroup. Heuristic searches were conducted with 1,000 bootstrap replicates (BS), tree bisection–reconnection (TBR) branch swapping, and simple stepwise addition. All characters had equal weight; gaps were treated as missing data.

Maximum likelihood trees with 1,000 BS were produced with IQ-tree 1.6.11 (Trifinopoulos et al., 2016). The best fit models for each dataset were calculated by ModelFinder (Kalyaanamoorthy et al., 2017). The K3Pu + F + R2 model was used for the complete plastid genome matrix. For the second matrix, the model of evolution was calculated for each sequence with IQ-tree 1.6.11 (Supplementary Table 1). The resulting trees were produced using the FigTree software v1.4.1^3^.

BI used MrBayes 3.2.7 (Ronquist and Huelsenbeck, 2003), and the best-fit model for each set was determined with the Bayesian information criterion (BIC) in jModeltest version 2.1.10 (Darriba et al., 2012; Supplementary Table 1). Four independent Markov chain Monte Carlo (MCMC) were run twice, each with 3,000,000 million generations. Trees were sampled every 1,000 generations, and the first 25% of these were discarded. The remaining trees were used to build the Bayesian tree of posterior probabilities.

## Results

### Plastid Genome Features

**FIGURE 1:**
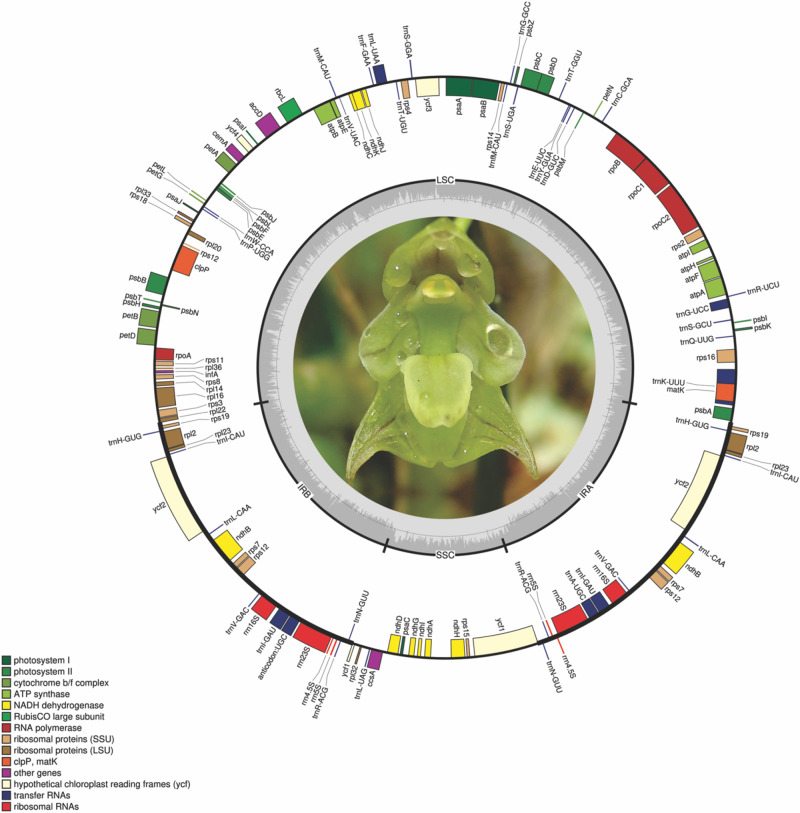
*Bulbophyllum regnellii* plastid genome. Genes shown on the outside of the circle are transcribed clockwise; genes on the inside are transcribed counterclockwise. The color of the gene boxes indicates the functional group to which the gene belongs. The thick lines indicate the length of the inverted repeats (IRA and IRB), separated by the large single-copy (LSC) and small single-copy (SSC) regions. The dashed darker gray area in the inner circle indicates genome GC content, whereas lighter gray area indicates AT content.

The eight *Bulbophyllum* plastid genomes were 146,401–151,493 bp long; the smallest was that of *Bulbophyllum plumosum* Barb.Rodr., whereas the largest was that of *Bulbophyllum regnellii* Rchb.f. The average coverage was between 45.26 and 247.55*x* for *B. epiphytum* and *B. steyermarkii* (Supplementary Table 2). The genomes have the typical angiosperm quadripartite structure (Figure 1 and Supplementary Figure 1): IRs = 25,465 bp (*Bulbophyllum granulosum* Barb.Rodr) to 26,340 bp (*Bulbophyllum mentosum* Barb.Robr), LSC = 82,354 bp (*B*. *epiphytum*) to 84,868 bp (*B*. *regnellii*), and SSC = 11,088 bp (*B. plumosum*) to 16,048 bp [*Bulbophyllum weddellii* (Lindl.) Rchb.f; Supplementary Figure 2]. GC content was similar (36.6–36.8%; Table 2).

**FIGURE 2:**
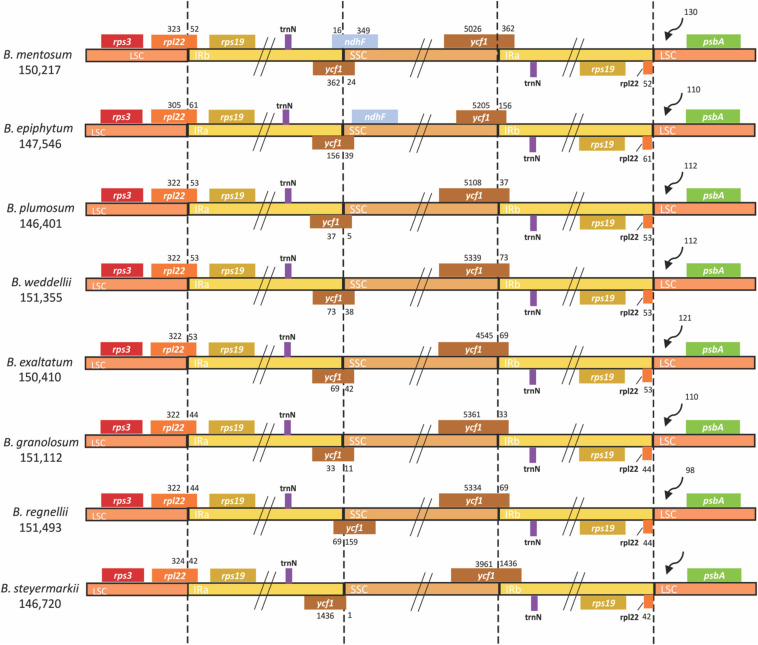
Comparison of LSC, IR, and SSC junctions in eight *Bulbophyllum* plastid genomes. Genes shown on the top are transcribed in reverse, and those shown underneath are forward. LSC, large single copy; IR, inverted repeated; SSC, small single copy.

For each plastid genome, 102 genes were predicted: 68 CDSs, 30 tRNAs, and four rDNAs. Eight CDSs, eight tRNAs, and all four rDNAs were present in the IRs (Supplementary Table 3). A small fragment of *ycf1* was found in IRb/SSC junction (Figure 2).

**FIGURE 3:**
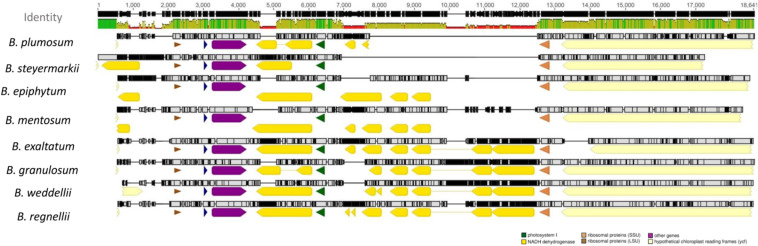
MAFFT alignment of the small single-copy (SSC) region of all plastid genomes. The yellow blocks represent the NDH genes. The green lines on the top represent the high similarity between the sequences, whereas red indicates the opposite. Genomes are organized in descending lengths.

Additionally, most *ndh* genes were truncated or completely lost. However, *ndhG* retained a full reading frame in *B. exaltatum*. Seven of the 11 *ndh* genes were located in the SSC, and their lengths were correlated with SSC expansion and contraction (*r* = 0.94, *p* ≤ 0.001). SSC expansion and contraction also determined the total plastid genome size (*r*^2^ = 0.89, *F*_1_, _6_ = 57.27, *p* ≤ 0.001). SSC length differences between the largest and smallest genomes were the 4,960 bp, in which the value corresponds to 97% of the total length differences among these plastid genomes (Figure 3).

In all plastid genomes, LSC/IRb and IRb/SSC junctions were in *rpl22* and *ycf1* (pseudogene), respectively, whereas the IRa/SSC and IRa/LSC junctions were in *ycf1* and *rpl22*. At the LSC/IRb border, IRb expanded up to 61 bp toward the *rpl22* gene and in the IRb/SSC border up to 1,436 bp toward the *ycf1* pseudogene. Furthermore, the IRb/SSC border of only *B. mentosum* was located in the *ndhF* pseudogene, with just 16 bp located in the IRb region. The *ndhF* pseudogene was close to but did not overlap with the IRb/SSC junction in *B. epiphytum* (Figure 2).

### Codon Usage

**FIGURE 4:**
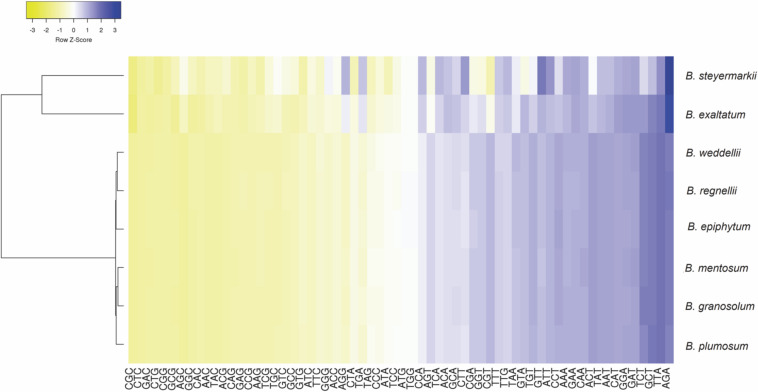
Relative synonymous codon usage ratio (RSCU) of protein-coding genes of the eight *Bulbophyllum* plastid genomes. Blue indicates a high RSCU ratio, and yellow indicates low.

Codon use frequency and RSCU assessed 68 unique CDSs among the eight plastid genomes. A range of 18,831–19,306 codons were examined, among which the most common codon was *AAA* (4.27%), whereas *CGC* (0.36%) was the rarest. *ATG* was the initiation codon for most protein-coding genes, but in the *rps19* (*GTG*) and *rpl2* genes (*ACG*), alternative initiation codons were found. Leucine was the most abundant amino acid, ranging between 1,258 and 2,687 codons in *B. mentosum* and *B. plumosum*, respectively, whereas cysteine was the least abundant, ranging between 209 and 776 codons in *B. exaltatum* and *B. mentosum*, respectively (Supplementary Table 4). RSCU analysis indicated that 29 of 32 codons ending with G/C were not frequently used, whereas 28 of 32 codons that ended with A/T had high RSCU (Figure 4 and Supplementary Table 5).

### Molecular Evolution Analysis of Protein-Coding Genes

**FIGURE 5:**
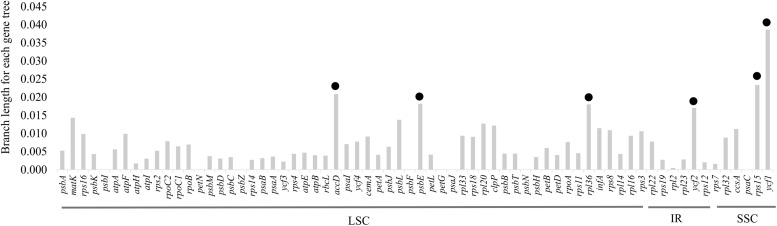
Molecular evolution of 68 protein-coding genes shared among the eight plastid genomes. The black circles indicate the six most divergent genes.

Analyses to investigate selection indicated that 89 putative sites were under positive selection. These sites were distributed in four out of 68 shared CDSs in these plastid genomes. The putatively most selected sites (adaptative selection) were found in *ycf1* (68 sites) followed by *ycf2*, *accD*, and *rps3* genes with 10, 10, and 1, respectively. Moreover, according to the average branch length of each protein-coding gene tree, the most divergent genes were *ycf1*, *rps15*, *accD*, *psbE*, *rpl36*, and *ycf2* (Figure 5).

### Characterization of Repeat Sequences and Simple Sequence Repeat

**FIGURE 6:**
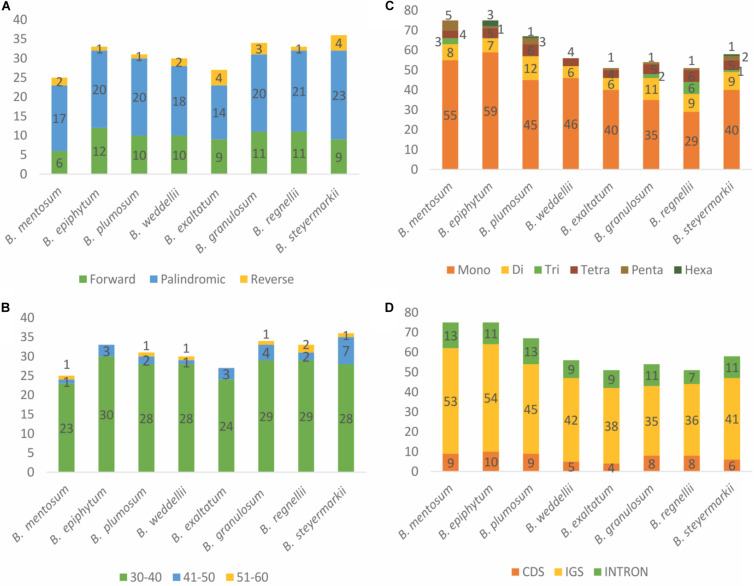
**(A)** Frequency of forward, reverse, and palindromic repeats. **(B)** Length frequency of tandem repeats. **(C)** Distribution and frequency of simple sequence repeat (SSR) types. **(D)** Distributions of SSRs for each region.

Forward, reverse, and palindromic repeats (>30 bp) were compared for the eight plastid genomes. In total, 249 repeats were identified in these plastid genomes, including 78 simple repeats, 153 palindromic repeats, and 18 reverse repeats (Figure 6A). *B. steyermarkii* exhibited the most (36), whereas *B. mentosum* had the fewest (25; Supplementary Table 6). The majority of repeat sequences were distributed in IGSs (54.62%), and the most frequent lengths were 30–40 bp (Figure 6B and Supplementary Table 7). Four palindromic repeats were shared by these eight plastid genomes (Table 3).

**TABLE 3:** Palindromic repeat sequences shared by *Bulbophyllum* species.

Size (bp)	Repeat sequence	Location
30	ATTTCAAAAAAAAATCGATTTTTTTTTGAA	Gene (*ccsA*)
37	TTATGTTTTGTATATTTGGATCAAATATACAAAACAT	IGS (*rps15-ycf1*)
30	GTCGGAGAGAGAGGGATTCGAACCCTCGGT	IGS [*trnS (GCU)*, *trnS (GGA)*]
32	CAAAAGGAGAGAGGGGGATTCGAACCCTCGAT	IGS [*trnS (UGA)*, *trnS (GGA*)]

*IGS, intergenic spacer.*

MISA analysis was performed to identify single SSRs in the plastid genomes. We detected 527 SSRs (Supplementary Table 8). The number of SSRs per species ranged from 51 to 75. Among these, there were 31–61 mononucleotide repeats, 8–14 dinucleotide repeats, 1–6 trinucleotide repeats, 5–7 tetranucleotide repeats, 1–5 pentanucleotide repeats, and 1–3 hexanucleotide repeats. Trinucleotide, pentanucleotide, and hexanucleotide repeats were absent from several plastid genomes (Figure 6C). Most SSRs were in the LSC (78%), followed by SSC (14%) and IR (8%). Most 527 SSRs were located in IGSs (347 SSRs), followed by CDS (95 SSRs) and introns (84 SSRs, Figure 6D). We designed 46 primer pairs for these microsatellites, which were present in at least four species (Supplementary Table 9).

### Hypervariable Regions

**FIGURE 7:**
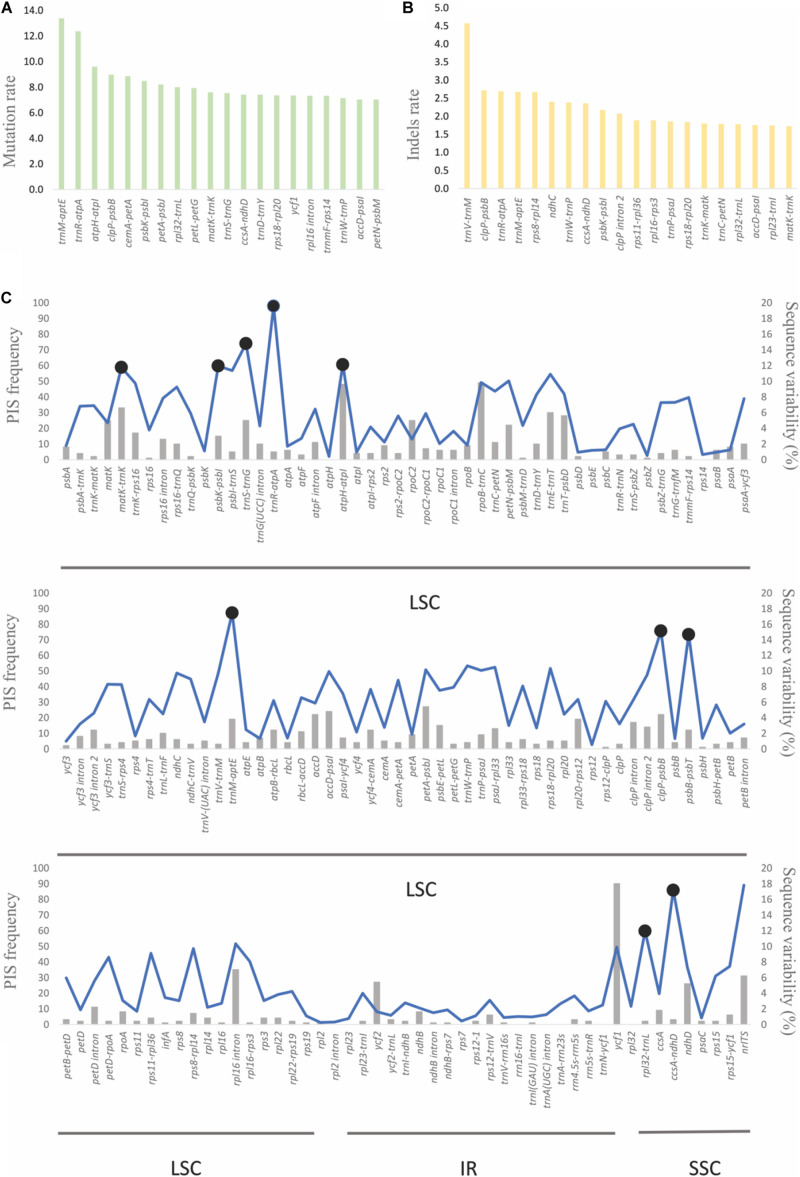
**(A)** The 20 sequences with the highest substitution and **(B)** indel rate. **(C)** Sequence variability and parsimony-informative sites (PIS) of the 148 plastid sequences and internal transcribed spacer (ITS). The blue line represents the sequence variability (SV), and the 10 most-variable regions are marked with black circles. The gray bars indicate the frequency of PIS of each sequence.

With the use of *B. steyermarkii* as a reference, the number of mutations and indels events were compared among these *Bulbophyllum* plastid genomes. There were 148 events (CDSs, IGSs, and introns) flanked by the same exon and longer than 150 bp: 4,064 mutations and 624 indels (Figures 7A,B). Intergenic spacers had the highest mutation rate, 5.21 mutations per 100 bp, following by introns and CDSs with 3.56 and 2.58, respectively (Table 4). Also, when indels were considered, IGSs had the most, 1.11 indels per 100 bp.

**TABLE 4:** Mutations, indel events, and mutation rates for each region type: protein coding genes (CDS), intergenic spacers (IGS), and introns.

Region	Mutations	Indels	Total length	Mutations/100 bp	Indels/100 bp
CDS	1,554	83	60,206	2.58	0.14
IGS	1,927	410	36,968	5.21	1.11
Intron	490	104	13,781	3.56	0.75

Furthermore, genes with the greatest number of potentially parsimony informative sites (PIS; Figure 7C) and greatest SV (Figure 7C, Supplementary Table 10; 15%, 184 of the total) were similar. Correlation analysis showed that SV was positively correlated with AT content (*r* = 0.81, *p* < 0.001). Primer pairs were developed for the 10 most-variable markers (Supplementary Table 11).

### Phylogenetic Analysis

Maximum parsimony, BI, and ML analyses were performed using (i) the complete plastid genomes of the eight *Bulbophyllum* species and (ii) the 10 most-variable regions; *D. aphyllum* was selected as the outgroup. The eight plastid genomes and outgroup were aligned in a single data matrix with a total of 163,916 nucleotide sites, of which 2,429 (1.48%) were identified as potentially PIS. The 10 most-variable regions matrix had 7,087 nucleotide sites with 285 (4%) potentially PIS (Figure 7C).

**FIGURE 8:**
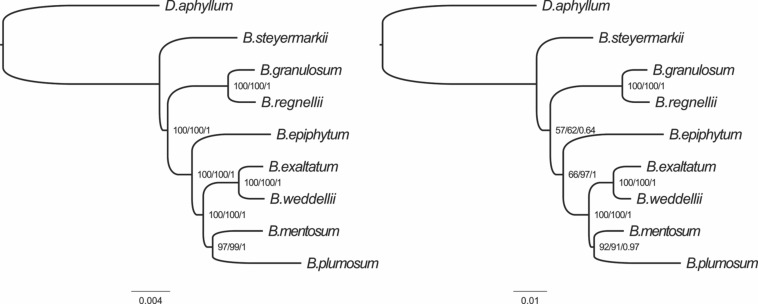
Phylogenetic relationships of the eight *Bulbophyllum* species constructed from whole genome **(left)** and the 10 most-variable regions **(right)** with maximum parsimony (MP), maximum likelihood (ML), and Bayesian inference (BI) methods. ML topology and branch length are represented. Numbers associated with branches are bootstrap percentages of MP/ML and BI posterior probabilities (in this order).

The tree topologies generated in the MP, BI, and ML analyses were identical for the two datasets. The majority of nodes received high support, but two nodes in the tree produced from the 10 most-variable regions had only moderate support (Figure 8).

## Discussion

### Plastid Genome Features

In this study, all eight plastid genomes of Neotropical *Bulbophyllum* species conserved the typical quadripartite structure previously reported in Orchidaceae (Zhitao et al., 2017), with exception of *Aphyllorchis montana* Rchb.f and *Gastrodia elata* Blume, heterotrophic orchids that have lost one IR (Feng et al., 2016; Yuan et al., 2018). Furthermore, gene arrangement and content were similar in the *Bulbophyllum* and *Dendrobium* (outgroup) genomes, with 68 CDS, 30 tRNA genes, and four ribosomal rRNA genes (Zhitao et al., 2017).

Although the general structure of *Bulbophyllum* plastid genomes is conserved, differences in *ndh* gene composition and total length were detected. The *ndh* family is composed of 11 genes involved in respiratory electron transport and chlororespiration (Ohyama et al., 1986; Martín and Sabater, 2010; Ueda et al., 2012). Ten of the 11 *ndh* genes in *Bulbophyllum* were deleted or truncated by point mutations or deletions, generating stop codons. A full reading frame for *ndhG* was observed only in *B. exaltatum*. Loss of *ndh* genes has been previously reported in orchids (Chang et al., 2006; Yang et al., 2013; Luo et al., 2014; Kim et al., 2015; Zhitao et al., 2017; Kim and Chase, 2017; Mauad et al., 2019; Smidt et al., 2020) and is useful in comparative analyses but not in phylogenetics owing to high levels of homoplasy depicted by the independent loss of some *ndh* genes in a number of species of Orchidaceae (Kim et al., 2015; Kim and Chase, 2017).

Previous studies concluded that the total length of angiosperm plastid genomes is influenced by contractions and expansions of the IRs (Chumley et al., 2006; Green, 2011; Weng et al., 2017). In contrast, here, we found that the length of *Bulbophyllum* plastid genomes is determined by the contraction and expansion of the SSC due to the independent loss of the seven *ndh* genes present in this region. The pronounced effect of the gain/loss of *ndh* genes in the SSC has been previously reported (Chang et al., 2006; Jheng et al., 2012; Kim et al., 2015; Zhu et al., 2018), but only in Zhitao et al. (2017) was the influence of LSC, IR, and SSC length on the genome size measured. As a result, they found that variation in LSC length plays an important role in plastid genome-size variation.

In general, gene content of the IR borders among *Bulbophyllum* orchids was similar. However, some differences in the IR/SSC junction were detected. In all *Bulbophyllum* plastid genomes, we identified the *trnH-rps19* gene cluster within the IR, and IR/LSC junctions were located 5’ of *rpl22*. In agreement with our findings, Luo et al. (2014); Kim et al. (2015), and others detected the same IR/LSC pattern among species of orchids, which is the type III of Wang et al. (2008) based on the *trnH-rps19* position among 123 angiosperms and suggests that this IR/LSC junction is one of those conserved among orchids.

However, different IR/SSC patterns have been reported in Orchidaceae (Yang et al., 2013; Luo et al., 2014; Niu et al., 2017a; Dong et al., 2018; Zhu et al., 2018). According to Kim et al. (2015), the stability of the IR/SSC junctions is highly affected by loss/gain of the *ndh* genes, especially *ndhF*, the absence of which could create complicated modifications like shifts in the IR border gene, length reduction of *ycf1* (less than 1 kb), and changes in *ycf1* position within the IR. Related to presence/absence of the *ndhF* pseudogene, three types of IR/SSC junctions were identified. The first was found in *B. epiphytum*, in which the *ndhF* pseudogene is present near IRb, but it does not overlap with the IRb/SSC junction. *B. mentosum* has the second type with the IRb expanded by 16 and 362 bp toward *ndhF* and *ycf1*, respectively. The other six genomes exhibit the third type in which the *ndhF* gene was lost, and the IR expands toward *ycf1* by 33–1,436 bp (Figure 2). Owing to the *Bulbophyllum* species with different IR border types not being grouped as in the phylogenetic tree, we suggest that IR borders do not have a simple phylogenetic signal.

### Codon Usage

Codon usage analysis revealed substitutions in the start codons of *rps19* and *rpl2* genes (putatively making these pseudogenes) and preferences for codons ending in A/T. These and RNA editing sites in *rps12* and *ycf15* genes have been previously reported in Orchidaceae (Chang et al., 2006; Yang et al., 2013; Luo et al., 2014; Barthet et al., 2015; Schelkunov et al., 2015), and they are probably involved in adaption in some manner (He et al., 2016). Additionally, RSCU analysis indicated that codons ending in A/T are more frequently used, similar to what has been found in most angiosperms (Meng et al., 2018; Tian et al., 2018; Raman et al., 2019), perhaps a result of compositional AT bias in plastid genomes (Morton, 1993; Zhou et al., 2008; Niu et al., 2017b).

### Molecular Evolution in Protein-Coding Genes

Protein-coding genes with sites putatively under positive selection, in general, occurred in the most divergent genes. Eighty-nine sites putatively under positive selection were located in *ycf1*, *ycf2*, *accD*, and *rps3*, the first being the second-largest plastid gene, essential for photosynthetic protein import (Kikuchi et al., 2013). Also, it is one of the most variable genes among the land plants, and therefore, it has been used in phylogenetic analyses (Dong et al., 2015). Other orchid plastid genomes studied (Niu et al., 2017b; Dong et al., 2018; Li et al., 2019) detected similar patterns.

Additionally, *ycf2* and *accD* had 10 putative sites under adaptative selection. The function of *ycf2* is still unknown, but *accD* regulates fatty acid synthesis, which is essential to maintain plastids (Kode et al., 2005). Additional study is needed to understand why these genes might be under adaptative selection.

### Characterization of Repeat Sequences and Simple Sequence Repeat

Repeat sequences may play an important role in genome recombination and rearrangements, size, and structure through illegitimate recombination and slipped-strand mispairing (Gemayel et al., 2010). In this study, 249 repeats were identified, the majority in non-coding regions. This result is similar to that of Yang et al. (2013), in which analyses of eight *Cymbidium* species found 232 repeat sequences, again mostly in non-coding regions. However, in *Cymbidium*, reverse repeats were the most abundant, whereas in *Bulbophyllum* palindromic repeats were the most common.

Owing to their high mutation rates (Gemayel et al., 2010), SSRs have been extensively used in populations genetic and phylogenetic studies (Kartzinel et al., 2013; Minasiewicz et al., 2018). Here, a total of 54 polymorphic SSRs were identified. The most abundant SSR type was the mononucleotide repeat (A/T), similar to the findings of Zhitao et al. (2017). Also, like other plastid genome studies (Zhang et al., 2016; Niu et al., 2017a), the majority of SSRs in *Bulbophyllum* is composed of polyT and polyA repeats (Supplementary Table 8). Owing to the great potential of plastid SSRs in genetic diversity studies, we provide here a complete set of *Bulbophyllum* microsatellite–primers pairs.

### Hypervariable Regions

As expected, the 148 sequences longer than 150 bp with the highest mutation and indel rates were located in non-coding regions, probably a result of their high AT content (Supplementary Figure 3). High AT content may contribute to replication errors creating mutations or deletions (Niu et al., 2017a). Based on number of mutations, indels, and conserved sites, we advocate use of 10 markers with barcoding and phylogenetic potential in *Bulbophyllum* (Figure 7). In recent years, numerous orchid plastid genomes have been sequenced. As a result, various plastid markers have been proposed for Orchidaceae (Yang et al., 2013; Niu et al., 2017a, c; Zhitao et al., 2017; Dong et al., 2018; Zhu et al., 2018; Li et al., 2019; Smidt et al., 2020). For instance, Niu et al. (2017c) suggested that *trnK-rps16*, *trnS-trnG*, and *rps16-trnQ* IGSs could be used for genera within Epidendroideae, and *clpP-psbB* and *rps16-trnQ* for Cypripedioideae. In addition, Niu et al. (2017a) recommended several for use in Apostasioideae. The identification of different highly variable markers among the orchid subfamilies suggests inconsistent divergence among taxa, a result corroborated here. Only four of the 10 most-variable markers identified for *Bulbophyllum* are shared with its sister genus, *Dendrobium* (*trnR-atpA*, *psbB-psbT*, *rpl32-trnL*, and *clpP-psbB*). Among the other six regions, two (*matK-trnK* and *trnS-trnG*) were identified by Niu et al.(2017a; 2017c) and Zhu et al. (2018), whereas four (*atpH-atpI*, *ccsA-ndhD*, *psbK-psbI*, and *trnM-aptE*) are reported for the first time here as highly variable in Orchidaceae.

Previous markers used in *Bulbophyllum* analyses proved here not to be highly variable; for example, Smidt et al. (2011) used *psbA-trnH* and *trnS-trnG* for the Neotropical sections of *Bulbophyllum*. Although, *trnS-trnG* had high statistics in this study, the SV of *psbA-trnH* was only 6.5%. Another example is Fischer et al. (2007), who studied Madagascan *Bulbophyllum* using four plastid IGSs (and nrITS). Among these four, one (*trnF-ndhJ*) was not considered in this study because it is missing from the plastid genomes of these Neotropical *Bulbophyllum* species. Use of markers with low SV is one of the reasons why in both previous studies some terminal nodes were unresolved and poorly supported.

In general, nrITS has been widely used in angiosperm phylogenetics, in particular in orchids (Fischer et al., 2007; Smidt et al., 2011, 2018), owing to its high variability and PIS. Here, we found one IGS, *trnR-atpA*, with greater variability than nrITS and two markers (*atpH-atpI* and *matK-trnK*) with more PIS than the nrITS but lower overall variability (Supplementary Figure 4). Thus, there are plastid markers with similar variability to nrITS that have been not thus far used in *Bulbophyllum* studies.

### Phylogenetic Analysis

We examined phylogenetic relationships among the eight *Bulbophyllum* species with the complete plastid genomes and the top 10 most-variable markers. Both datasets produced the same topology with similar support for all three phylogenetic methods. Also, this tree is congruent with that of Neotropical *Bulbophyllum* in Smidt et al. (2011). Furthermore, in Smidt et al. (2011), the position of *B. mentosum* was unclear. In the nrITS tree, *B. mentosum* was a member of *Bulbophyllum* section *Micranthae*, but in the plastid tree from that analysis, it should be in *Bulbophyllum* section *Xiphizusa*, agreeing with results here for both matrices analyzed. The incongruence between nuclear and plastid data found in Smidt et al. (2011) may be a result of ancient hybridization (Barber et al., 2007).

## Conclusion

In summary, we sequenced here eight complete plastomes of *Bulbophyllum* species, representing five of the six Neotropical taxonomic sections. In general, the plastomes were similar in gene content and structure, except for *ndh* gene composition. However, despite this general high similarity, we detected several regions with higher variability than the nuclear and plastid molecular markers previously used in this genus. We have thus provided important molecular resources for *Bulbophyllum*, comprising 10 highly variable regions and 54 microsatellites (and primers to amplify them). These molecular resources for *Bulbophyllum* will be useful to improve our understanding of phylogenic relationships, population genetics, and phylogeography and aid in species identification.

## Data Availability Statement

The datasets presented in this study can be found in online repositories. The names of the repository/repositories and accession number(s) can be found below: https://www.ncbi.nlm.nih.gov/genbank/, MN604056, MN737573, MN580547, MN604059, MN604054, MN604055, MN604057, MN604058.

## Author Contributions

ECS designed the study. MCC collected specimens. MZ-P, LV, VB, EB and ES performed the laboratory work. MZ-P and LV performed all analyses. All authors contributed to writing of the manuscript.

## Conflict of Interest

The authors declare that the research was conducted in the absence of any commercial or financial relationships that could be construed as a potential conflict of interest.

## Footnotes

1 https://www.qiagenbioinformatics.com/

2 http://bioinfo.ut.ee/primer3-0.4.0/

3 http://tree.bio.ed.ac.uk
