# Supplementary material for: Comparative Plastid Genomics of Neotropical Bulbophyllum (Orchidaceae; Epidendroideae)
Source: Front Plant Sci. 2020 Jul 3;11:799. doi: 10.3389/fpls.2020.00799 (PMC7347972; doi:10.3389/fpls.2020.00799)

# *Supplementary Material*

## Comparative plastid genome of Neotropical *Bulbophyllum* Thouars (Orchidaceae; Epidendroideae)

**Michelle Zavala Paez[1*], Leila do Nascimento Vieira[1], Valter Antônio de Baura[2], Eduardo Balsanelli[2], Emanuel Maltempi de Souza[2], Marco Cerna Cevallos[3,] Mark W. Chase[4,5], Eric de Camargo Smidt[1*]**

*** Correspondence:** *michellezavalapaez@outlook.es*; *ecsmidt@gmail.com*

**Supplementary Table 1.** The best-fit model chosen for phylogenetic analysis for each set and sequence.

| **Set** | **Sequences** | **Bayesian inference** | **Maximum likelihood** |
|---|---|---|---|
| Whole genome | | GTR+I+G | K3Pu+F+R2 |
| Top 10 variable sequences | *matK-trnK* | GTR+G | K3Pu+F+G4 |
| | *atpH-atpI* | GTR | K3Pu+F |
| | *ccsA-ndhD* | F81+G | K3Pu+F+I |
| | *clpP-psbB* | F81+G | F81+F+G4 |
| | *psbB-psbT* | GTR+G | K3Pu+F+I |
| | *psbK-psbI* | GTR | K3Pu+F |
| | *rpl32-trnL* | HKY | K3Pu+F |
| | *trnM-atpE* | F81 | F81+F |
| | *trnR-atpA* | F81 | K3Pu+F |
| | *trnS-trnG* | GTR+G | K3Pu+F+G4 |

**Supplementary Table 2.** Summary of number of reads used and genome coverage of the assembly.

| Species | Total reads | Total plastid reads | Average coverage | Standard deviation | % of plastid reads |
|---|---|---|---|---|---|
| ***B. mentosum*** | 718,275 | 184,119 | 224.17 | 82.83 | 25,63 |
| ***B. epiphytum*** | 251,342 | 31,075 | 45.26 | 19.2 | 12,36 |
| ***B. plumosum*** | 1,129,148 | 205,114 | 235.29 | 71.09 | 18,17 |
| ***B. weddellii*** | 849,584 | 94,326 | 133.38 | 65.76 | 11,10 |
| ***B. exaltatum*** | 685,933 | 62,715 | 85.94 | 51.15 | 9,14 |
| ***B. granulosum*** | 289,202 | 131,809 | 194.7 | 69.43 | 45,58 |
| ***B. regnellii*** | 612,812 | 153,311 | 148.57 | 44.61 | 25,02 |
| ***B. steyermarkii*** | 453,955 | 186,928 | 247.55 | 101.21 | 41,18 |

**Supplementary Table 3.** A list of genes found in the plastid genomes of eight *Bulbophyllum* species.

| **Function** | **Gene group** | **Gene name** |
|---|---|---|
| Photosynthesis pathways | Photosystem I | *psa* A, B, C, I, J |
| | Photosystem I assembly | *ycf* 3**,4 |
| | Photosystem II | *psb A, B, C, D, E, F, G, H, I, J, K, L, M, N, T, Z* |
| | F-type ATP synthase | *atp* A, B, E, F*, H, I |
| | NAD(P)H-dehydrogenase Complex | *ndh*G |
| | Component of cytochrome b6/f Complex | *pet* A, B*, D*, G, L, N |
| | Cytochrome c biogenesis protein | *ccs*A |
| | Large subunit of Rubisco | *rbcL* |
| Structural RNAs | Transfer RNAs | *trn* A-UGC*▪, C-GCA, D-GUC, E-UUC, F-GAA, fM-CAU, G-GCC, G-UCC▪, H-GUG, I-CAU▪, I-GAU*▪, K-UUU*, L-CAA▪, L-UAA*, L-UAG, M-CAU, N-GUU▪, P-UGG, Q-UUG, R-ACG▪, R-UCU, S-GCU, S-GGA, S-UGA, T-GGU, T-UGU, V-GAC▪, V-UAC*, W-CCA, Y-GUA |
| | Ribosomal RNAs | *rrn 4.5▪, 5▪, 16▪, 23▪* |
| Transcription and translation related genes | Transcription | *rpo* A, B, C1*, C2 |
| | Ribosomal proteins | *rps* 11, 12**▪+, 14, 15, 16*, 18, 19▪, 2, 3, 4, 7▪, 8; rpl 2*▪. 14, 16*, 20, 22▪, 23▪, 32, 33, 36 |
| | Translation initiation factor | *inf*A |
| Other genes | RNA processing | *mat*K |
| | Proteolysis | *clp*P ** |
| | Fatty acid synthesis | *acc*D |
| | Carbon metabolism | *cem*A |
| Unknown Proteins | Conserved reading frames | *ycf* 1▪*, 2▪ |
| Pseudogenes | | *ndh*A, B**▪, C, D, E, F, H, I, J, K; one copy of *ycf*1 |

*, 1 intron in gene; **, 2 introns in gene; ▪, gene repeated in IR region; +, gene has 2 separate transcription units.

**Supplementary Table 4.** The codon usage frequency of 68 CDS shared among the eight *Bulbophyllum* plastid genomes.

| Amino acid | Codon | % of AA | | | | | | | | Count | | | | | | | |
|---|---|---|---|---|---|---|---|---|---|---|---|---|---|---|---|---|---|
| | | ***B.men*** | ***B. epi*** | ***B.plu*** | ***B.wed*** | ***B.exa*** | ***B.gra*** | ***B.reg*** | ***B.est*** | ***B.men*** | ***B. epi*** | ***B.plu*** | ***B.wed*** | ***B.exa*** | ***B.gra*** | ***B.reg*** | ***B.est*** |
| Ala | GCA | 0.71% | 1.61% | 0.66% | 0.64% | 1.69% | 1.66% | 1.67% | 0.76% | 136 | 309 | 126 | 124 | 321 | 321 | 323 | 144 |
| Ala | GCC | 0.45% | 0.68% | 0.35% | 0.35% | 0.74% | 0.74% | 0.74% | 0.45% | 87 | 131 | 67 | 67 | 141 | 143 | 143 | 85 |
| Ala | GCG | 0.39% | 0.55% | 0.40% | 0.42% | 0.51% | 0.51% | 0.48% | 0.40% | 74 | 105 | 77 | 81 | 97 | 99 | 93 | 75 |
| Ala | GCT | 0.51% | 2.48% | 0.38% | 0.37% | 2.57% | 2.53% | 2.53% | 0.61% | 97 | 475 | 73 | 72 | 489 | 489 | 488 | 114 |
| Arg | AGA | 3.32% | 1.98% | 1.44% | 1.48% | 1.91% | 1.94% | 1.96% | 3.16% | 636 | 379 | 276 | 286 | 364 | 374 | 379 | 595 |
| Arg | AGG | 2.08% | 0.65% | 1.08% | 1.09% | 0.66% | 0.65% | 0.64% | 2.06% | 398 | 124 | 207 | 211 | 125 | 126 | 123 | 388 |
| Arg | CGA | 1.14% | 1.33% | 0.63% | 0.62% | 1.42% | 1.39% | 1.39% | 1.19% | 219 | 254 | 120 | 120 | 270 | 269 | 269 | 225 |
| Arg | CGC | 0.48% | 0.36% | 0.32% | 0.31% | 0.33% | 0.33% | 0.34% | 0.47% | 92 | 68 | 62 | 60 | 63 | 64 | 65 | 89 |
| Arg | CGG | 0.71% | 0.43% | 0.84% | 0.84% | 0.39% | 0.40% | 0.40% | 0.73% | 137 | 82 | 162 | 162 | 74 | 77 | 77 | 138 |
| Arg | CGT | 0.68% | 1.49% | 0.36% | 0.35% | 1.51% | 1.50% | 1.51% | 0.75% | 130 | 285 | 69 | 68 | 288 | 290 | 291 | 141 |
| Asn | AAC | 1.73% | 1.01% | 1.71% | 1.72% | 0.96% | 0.95% | 0.95% | 1.62% | 331 | 193 | 329 | 331 | 183 | 184 | 183 | 306 |
| Asn | AAT | 3.74% | 3.92% | 2.67% | 2.67% | 3.79% | 3.83% | 3.84% | 3.77% | 718 | 750 | 513 | 514 | 721 | 739 | 740 | 709 |
| Asp | GAC | 0.70% | 0.75% | 0.89% | 0.89% | 0.77% | 0.77% | 0.76% | 0.70% | 135 | 144 | 171 | 172 | 146 | 148 | 146 | 132 |
| Asp | GAT | 2.29% | 3.30% | 1.77% | 1.75% | 3.28% | 3.29% | 3.30% | 2.41% | 440 | 632 | 339 | 338 | 624 | 635 | 636 | 454 |
| Cys | TGC | 1.95% | 0.34% | 0.81% | 0.81% | 0.26% | 0.26% | 0.26% | 1.92% | 374 | 66 | 155 | 156 | 50 | 51 | 50 | 361 |
| Cys | TGT | 2.10% | 0.98% | 0.83% | 0.83% | 0.84% | 0.84% | 0.85% | 2.06% | 402 | 187 | 159 | 160 | 159 | 163 | 164 | 388 |
| Gln | CAA | 1.69% | 2.82% | 2.09% | 2.09% | 2.84% | 2.85% | 2.85% | 1.86% | 324 | 539 | 402 | 402 | 540 | 551 | 549 | 350 |
| Gln | CAG | 0.78% | 0.83% | 1.66% | 1.65% | 0.84% | 0.85% | 0.86% | 0.77% | 149 | 159 | 319 | 319 | 160 | 164 | 165 | 145 |
| Glu | GAA | 2.24% | 4.23% | 2.36% | 2.42% | 4.26% | 4.25% | 4.27% | 2.34% | 430 | 809 | 453 | 466 | 810 | 820 | 823 | 440 |
| Glu | GAG | 0.91% | 1.31% | 1.81% | 1.79% | 1.33% | 1.35% | 1.36% | 0.97% | 174 | 250 | 347 | 345 | 253 | 261 | 263 | 183 |
| Gly | GGA | 1.82% | 2.67% | 1.26% | 1.27% | 2.70% | 2.69% | 2.70% | 1.83% | 349 | 511 | 242 | 244 | 513 | 519 | 521 | 345 |
| Gly | GGC | 0.97% | 0.71% | 0.49% | 0.49% | 0.72% | 0.70% | 0.69% | 0.97% | 186 | 136 | 95 | 94 | 137 | 135 | 134 | 183 |
| Gly | GGG | 1.53% | 1.09% | 1.33% | 1.32% | 1.12% | 1.12% | 1.11% | 1.52% | 293 | 209 | 255 | 254 | 214 | 216 | 214 | 287 |
| Gly | GGT | 0.92% | 2.29% | 0.80% | 0.80% | 2.28% | 2.27% | 2.27% | 0.97% | 176 | 438 | 153 | 155 | 434 | 439 | 438 | 183 |

**Supplementary Table 4.** Cont.

| Amino acid | Codon | % of AA | | | | | | | | Count | | | | | | | |
|---|---|---|---|---|---|---|---|---|---|---|---|---|---|---|---|---|---|
| | | ***B.men*** | ***B. epi*** | ***B.plu*** | ***B.wed*** | ***B.exa*** | ***B.gra*** | ***B.reg*** | ***B.est*** | ***B.men*** | ***B. epi*** | ***B.plu*** | ***B.wed*** | ***B.exa*** | ***B.gra*** | ***B.reg*** | ***B.est*** |
| His | CAC | 0.65% | 0.54% | 0.82% | 0.84% | 0.55% | 0.53% | 0.52% | 0.64% | 124 | 104 | 157 | 161 | 104 | 103 | 101 | 121 |
| His | CAT | 1.58% | 2.11% | 1.31% | 1.32% | 2.04% | 2.02% | 2.01% | 1.68% | 303 | 403 | 252 | 255 | 388 | 390 | 388 | 317 |
| Ile | ATA | 1.37% | 2.59% | 3.41% | 3.36% | 2.57% | 2.60% | 2.58% | 1.46% | 262 | 495 | 654 | 648 | 490 | 502 | 497 | 274 |
| Ile | ATC | 1.73% | 1.56% | 2.36% | 2.36% | 1.62% | 1.61% | 1.61% | 1.70% | 331 | 298 | 454 | 454 | 308 | 311 | 311 | 321 |
| Ile | ATT | 3.34% | 4.17% | 2.88% | 2.84% | 4.13% | 4.15% | 4.16% | 3.44% | 640 | 798 | 552 | 548 | 785 | 802 | 803 | 647 |
| Leu | CTA | 0.65% | 1.35% | 2.21% | 2.25% | 1.38% | 1.38% | 1.40% | 0.69% | 124 | 259 | 425 | 434 | 263 | 267 | 271 | 130 |
| Leu | CTC | 0.49% | 0.60% | 1.66% | 1.65% | 0.59% | 0.60% | 0.60% | 0.50% | 93 | 114 | 318 | 319 | 112 | 115 | 115 | 95 |
| Leu | CTG | 0.46% | 0.64% | 1.98% | 1.98% | 0.68% | 0.69% | 0.68% | 0.45% | 88 | 122 | 380 | 381 | 130 | 133 | 131 | 85 |
| Leu | CTT | 1.71% | 2.03% | 2.43% | 2.46% | 1.99% | 1.99% | 1.99% | 1.74% | 328 | 388 | 466 | 474 | 379 | 384 | 383 | 327 |
| Leu | TTA | 1.71% | 3.11% | 2.36% | 2.25% | 3.23% | 3.25% | 3.25% | 1.85% | 327 | 596 | 454 | 434 | 615 | 627 | 626 | 348 |
| Leu | TTG | 1.55% | 2.16% | 3.35% | 3.34% | 2.14% | 2.15% | 2.15% | 1.68% | 298 | 413 | 644 | 643 | 407 | 415 | 415 | 316 |
| Lys | AAA | 4.05% | 4.37% | 4.30% | 4.28% | 4.36% | 4.43% | 4.40% | 4.03% | 777 | 837 | 826 | 826 | 829 | 856 | 848 | 759 |
| Lys | AAG | 1.69% | 1.48% | 3.17% | 3.25% | 1.39% | 1.40% | 1.40% | 1.61% | 324 | 284 | 609 | 627 | 264 | 270 | 270 | 303 |
| Met | ATG | 0.98% | 2.20% | 2.86% | 2.86% | 2.25% | 2.28% | 2.29% | 1.05% | 188 | 420 | 549 | 551 | 428 | 441 | 442 | 197 |
| Phe | TTC | 2.52% | 1.98% | 2.89% | 2.89% | 1.98% | 1.95% | 1.94% | 2.39% | 483 | 379 | 554 | 558 | 376 | 376 | 375 | 451 |
| Phe | TTT | 4.69% | 3.66% | 3.34% | 3.33% | 3.60% | 3.59% | 3.62% | 4.48% | 900 | 701 | 642 | 642 | 685 | 694 | 698 | 844 |
| Pro | CCA | 0.98% | 1.08% | 1.30% | 1.29% | 1.09% | 1.08% | 1.07% | 0.92% | 187 | 207 | 249 | 248 | 208 | 208 | 207 | 174 |
| Pro | CCC | 0.58% | 0.85% | 0.66% | 0.68% | 0.91% | 0.88% | 0.89% | 0.64% | 112 | 162 | 126 | 132 | 173 | 169 | 171 | 121 |
| Pro | CCG | 0.49% | 0.44% | 0.92% | 0.93% | 0.48% | 0.46% | 0.47% | 0.47% | 93 | 84 | 176 | 179 | 91 | 89 | 90 | 88 |
| Pro | CCT | 0.74% | 1.57% | 0.85% | 0.83% | 1.60% | 1.57% | 1.58% | 0.79% | 141 | 300 | 164 | 160 | 304 | 304 | 305 | 148 |
| Ser | AGC | 1.97% | 0.42% | 0.37% | 0.38% | 0.39% | 0.39% | 0.39% | 1.91% | 378 | 81 | 71 | 74 | 74 | 76 | 75 | 359 |
| Ser | AGT | 1.81% | 1.65% | 0.60% | 0.59% | 1.60% | 1.58% | 1.59% | 1.78% | 348 | 315 | 116 | 114 | 304 | 305 | 307 | 335 |
| Ser | TCA | 2.63% | 1.38% | 1.46% | 1.49% | 1.45% | 1.46% | 1.46% | 2.51% | 505 | 265 | 281 | 287 | 275 | 281 | 282 | 472 |
| Ser | TCC | 1.79% | 1.23% | 1.12% | 1.12% | 1.18% | 1.17% | 1.14% | 1.75% | 344 | 236 | 215 | 216 | 225 | 225 | 219 | 329 |
| Ser | TCG | 1.66% | 0.60% | 0.95% | 0.95% | 0.60% | 0.61% | 0.61% | 1.59% | 319 | 115 | 183 | 183 | 114 | 117 | 118 | 299 |
| Ser | TCT | 2.25% | 2.25% | 1.49% | 1.51% | 2.31% | 2.32% | 2.30% | 2.30% | 432 | 431 | 287 | 291 | 440 | 447 | 443 | 433 |
| Stp | TAA | 2.45% | 0.19% | 2.23% | 2.22% | 0.17% | 0.16% | 0.17% | 2.22% | 469 | 37 | 428 | 428 | 32 | 31 | 32 | 418 |

**Supplementary Table 4.** Cont.

| Amino acid | Codon | % of AA | | | | | | | | Count | | | | | | | |
|---|---|---|---|---|---|---|---|---|---|---|---|---|---|---|---|---|---|
| | | ***B.men*** | ***B. epi*** | ***B.plu*** | ***B.wed*** | ***B.exa*** | ***B.gra*** | ***B.reg*** | ***B.est*** | ***B.men*** | ***B. epi*** | ***B.plu*** | ***B.wed*** | ***B.exa*** | ***B.gra*** | ***B.reg*** | ***B.est*** |
| Stp | TAG | 1.31% | 0.13% | 2.61% | 2.58% | 0.11% | 0.10% | 0.10% | 1.22% | 252 | 25 | 501 | 498 | 20 | 20 | 20 | 230 |
| Stp | TGA | 2.64% | 0.10% | 1.89% | 1.95% | 0.08% | 0.09% | 0.08% | 2.42% | 507 | 20 | 363 | 376 | 16 | 17 | 16 | 455 |
| Thr | ACA | 1.77% | 1.51% | 1.12% | 1.13% | 1.52% | 1.51% | 1.50% | 1.77% | 339 | 288 | 215 | 217 | 289 | 291 | 290 | 334 |
| Thr | ACC | 1.16% | 0.90% | 0.63% | 0.62% | 0.92% | 0.91% | 0.90% | 1.16% | 223 | 172 | 121 | 119 | 176 | 176 | 174 | 219 |
| Thr | ACG | 1.06% | 0.53% | 0.64% | 0.66% | 0.53% | 0.54% | 0.54% | 1.00% | 203 | 102 | 123 | 127 | 100 | 104 | 104 | 188 |
| Thr | ACT | 1.24% | 2.04% | 0.68% | 0.68% | 2.08% | 2.06% | 2.07% | 1.32% | 237 | 391 | 131 | 131 | 396 | 398 | 399 | 249 |
| Trp | TGG | 2.49% | 1.78% | 1.99% | 2.02% | 1.77% | 1.77% | 1.77% | 2.38% | 477 | 340 | 383 | 390 | 336 | 341 | 341 | 449 |
| Tyr | TAC | 1.77% | 0.75% | 1.67% | 1.66% | 0.70% | 0.68% | 0.72% | 1.66% | 340 | 144 | 320 | 320 | 133 | 132 | 139 | 313 |
| Tyr | TAT | 3.64% | 2.87% | 2.30% | 2.28% | 2.75% | 2.79% | 2.79% | 3.58% | 697 | 550 | 441 | 439 | 523 | 539 | 538 | 675 |
| Val | GTA | 0.50% | 1.90% | 1.81% | 1.79% | 2.02% | 1.98% | 1.97% | 0.62% | 95 | 364 | 347 | 345 | 385 | 383 | 380 | 116 |
| Val | GTC | 0.59% | 0.67% | 1.28% | 1.26% | 0.69% | 0.71% | 0.72% | 0.62% | 114 | 128 | 245 | 242 | 132 | 138 | 138 | 116 |
| Val | GTG | 0.36% | 0.86% | 1.39% | 1.38% | 0.89% | 0.89% | 0.90% | 0.42% | 69 | 164 | 266 | 267 | 169 | 172 | 174 | 79 |
| Val | GTT | 1.13% | 1.91% | 1.78% | 1.76% | 1.98% | 1.97% | 1.95% | 1.26% | 216 | 366 | 341 | 339 | 376 | 380 | 377 | 237 |

* B. men: *B. mentosum*, B. epi: B. *epiphytum*, B: plu: B. *plumosum,* B. wed: *B. weddellii*, B. exa: *B. exaltatum*, B. gra: *B. granulosum,* B. reg: *B. regnellii*, B. ste: *B. steyermarkii*

**Supplementary Table 5.** The relative synonymous codon usage (RSCU) of 68 CDS shared among the eight *Bulbophyllum* plastid genomes.

| Amino acid | Codon | RSCU | | | | | | | |
|---|---|---|---|---|---|---|---|---|---|
| | | ***B.men*** | ***B. epi*** | ***B.plu*** | ***B.wed*** | ***B.exa*** | ***B.gra*** | ***B.reg*** | ***B.est*** |
| Ala | GCA | 1.38 | 1.21 | 1.47 | 1.44 | 1.23 | 1.22 | 1.23 | 1.38 |
| Ala | GCC | 0.88 | 0.51 | 0.78 | 0.78 | 0.54 | 0.54 | 0.55 | 0.81 |
| Ala | GCG | 0.75 | 0.41 | 0.90 | 0.94 | 0.37 | 0.38 | 0.36 | 0.72 |
| Ala | GCT | 0.98 | 1.86 | 0.85 | 0.84 | 1.87 | 1.86 | 1.86 | 1.09 |
| Arg | AGA | 2.37 | 1.91 | 1.85 | 1.89 | 1.84 | 1.87 | 1.89 | 2.27 |
| Arg | AGG | 1.48 | 0.62 | 1.39 | 1.40 | 0.63 | 0.63 | 0.61 | 1.48 |
| Arg | CGA | 0.82 | 1.28 | 0.80 | 0.79 | 1.37 | 1.35 | 1.34 | 0.86 |
| Arg | CGC | 0.34 | 0.34 | 0.42 | 0.40 | 0.32 | 0.32 | 0.32 | 0.34 |
| Arg | CGG | 0.51 | 0.41 | 1.08 | 1.07 | 0.38 | 0.39 | 0.38 | 0.53 |
| Arg | CGT | 0.48 | 1.43 | 0.46 | 0.45 | 1.46 | 1.45 | 1.45 | 0.54 |
| Asn | AAC | 0.63 | 0.41 | 0.78 | 0.78 | 0.40 | 0.40 | 0.40 | 0.60 |
| Asn | AAT | 1.37 | 1.59 | 1.22 | 1.22 | 1.60 | 1.60 | 1.60 | 1.40 |
| Asp | GAC | 0.47 | 0.37 | 0.67 | 0.67 | 0.38 | 0.38 | 0.37 | 0.45 |
| Asp | GAT | 1.53 | 1.63 | 1.33 | 1.33 | 1.62 | 1.62 | 1.63 | 1.55 |
| Cys | TGC | 0.96 | 0.52 | 0.99 | 0.99 | 0.48 | 0.48 | 0.47 | 0.96 |
| Cys | TGT | 1.04 | 1.48 | 1.01 | 1.01 | 1.52 | 1.52 | 1.53 | 1.04 |
| Gln | CAA | 1.37 | 1.54 | 1.12 | 1.12 | 1.54 | 1.54 | 1.54 | 1.41 |
| Gln | CAG | 0.63 | 0.46 | 0.88 | 0.88 | 0.46 | 0.46 | 0.46 | 0.59 |
| Glu | GAA | 1.42 | 1.53 | 1.13 | 1.15 | 1.52 | 1.52 | 1.52 | 1.41 |
| Glu | GAG | 0.58 | 0.47 | 0.87 | 0.85 | 0.48 | 0.48 | 0.48 | 0.59 |
| Gly | GGA | 1.39 | 1.58 | 1.30 | 1.31 | 1.58 | 1.59 | 1.59 | 1.38 |
| Gly | GGC | 0.74 | 0.42 | 0.51 | 0.50 | 0.42 | 0.41 | 0.41 | 0.73 |
| Gly | GGG | 1.17 | 0.65 | 1.37 | 1.36 | 0.66 | 0.66 | 0.65 | 1.15 |
| Gly | GGT | 0.70 | 1.35 | 0.82 | 0.83 | 1.34 | 1.34 | 1.34 | 0.73 |
| His | CAC | 0.58 | 0.41 | 0.77 | 0.77 | 0.42 | 0.42 | 0.41 | 0.55 |
| His | CAT | 1.42 | 1.59 | 1.23 | 1.23 | 1.58 | 1.58 | 1.59 | 1.45 |
| Ile | ATA | 0.64 | 0.93 | 1.18 | 1.18 | 0.93 | 0.93 | 0.93 | 0.66 |
| Ile | ATC | 0.81 | 0.56 | 0.82 | 0.83 | 0.58 | 0.58 | 0.58 | 0.78 |
| Ile | ATT | 1.56 | 1.50 | 1.00 | 1.00 | 1.49 | 1.49 | 1.50 | 1.56 |
| Leu | CTA | 0.59 | 0.82 | 0.95 | 0.97 | 0.83 | 0.83 | 0.84 | 0.60 |
| Leu | CTC | 0.44 | 0.36 | 0.71 | 0.71 | 0.35 | 0.36 | 0.36 | 0.44 |
| Leu | CTG | 0.42 | 0.39 | 0.85 | 0.85 | 0.41 | 0.41 | 0.40 | 0.39 |
| Leu | CTT | 1.56 | 1.23 | 1.04 | 1.06 | 1.19 | 1.19 | 1.18 | 1.51 |
| Leu | TTA | 1.56 | 1.89 | 1.01 | 0.97 | 1.94 | 1.94 | 1.94 | 1.60 |
| Leu | TTG | 1.42 | 1.31 | 1.44 | 1.44 | 1.28 | 1.28 | 1.28 | 1.46 |

**Supplementary Table 5.** Cont.

| Amino acid | Codon | RSCU | | | | | | | |
|---|---|---|---|---|---|---|---|---|---|
| | | ***B.men*** | ***B. epi*** | ***B.plu*** | ***B.wed*** | ***B.exa*** | ***B.gra*** | ***B.reg*** | ***B.est*** |
| Lys | AAA | 1.41 | 1.49 | 1.15 | 1.14 | 1.52 | 1.52 | 1.52 | 1.43 |
| Lys | AAG | 0.59 | 0.51 | 0.85 | 0.86 | 0.48 | 0.48 | 0.48 | 0.57 |
| Met | ATG | 1.00 | 1.00 | 1.00 | 1.00 | 1.00 | 1.00 | 1.00 | 1.00 |
| Phe | TTC | 0.70 | 0.70 | 0.93 | 0.93 | 0.71 | 0.70 | 0.70 | 0.70 |
| Phe | TTT | 1.30 | 1.30 | 1.07 | 1.07 | 1.29 | 1.30 | 1.30 | 1.30 |
| Pro | CCA | 1.40 | 1.10 | 1.39 | 1.38 | 1.07 | 1.08 | 1.07 | 1.31 |
| Pro | CCC | 0.84 | 0.86 | 0.70 | 0.73 | 0.89 | 0.88 | 0.88 | 0.91 |
| Pro | CCG | 0.70 | 0.45 | 0.98 | 1.00 | 0.47 | 0.46 | 0.47 | 0.66 |
| Pro | CCT | 1.06 | 1.59 | 0.92 | 0.89 | 1.57 | 1.58 | 1.58 | 1.11 |
| Ser | AGC | 0.98 | 0.34 | 0.37 | 0.38 | 0.31 | 0.31 | 0.31 | 0.97 |
| Ser | AGT | 0.90 | 1.31 | 0.60 | 0.59 | 1.27 | 1.26 | 1.28 | 0.90 |
| Ser | TCA | 1.30 | 1.10 | 1.46 | 1.48 | 1.15 | 1.16 | 1.17 | 1.27 |
| Ser | TCC | 0.89 | 0.98 | 1.12 | 1.11 | 0.94 | 0.93 | 0.91 | 0.89 |
| Ser | TCG | 0.82 | 0.48 | 0.95 | 0.94 | 0.48 | 0.48 | 0.49 | 0.81 |
| Ser | TCT | 1.11 | 1.79 | 1.49 | 1.50 | 1.84 | 1.85 | 1.84 | 1.17 |
| Stp | TAA | 1.15 | 1.35 | 0.99 | 0.99 | 1.41 | 1.37 | 1.41 | 1.14 |
| Stp | TAG | 0.62 | 0.91 | 1.16 | 1.15 | 0.88 | 0.88 | 0.88 | 0.63 |
| Stp | TGA | 1.24 | 0.73 | 0.84 | 0.87 | 0.71 | 0.75 | 0.71 | 1.24 |
| Thr | ACA | 1.35 | 1.21 | 1.46 | 1.46 | 1.20 | 1.20 | 1.20 | 1.35 |
| Thr | ACC | 0.89 | 0.72 | 0.82 | 0.80 | 0.73 | 0.73 | 0.72 | 0.88 |
| Thr | ACG | 0.81 | 0.43 | 0.83 | 0.86 | 0.42 | 0.43 | 0.43 | 0.76 |
| Thr | ACT | 0.95 | 1.64 | 0.89 | 0.88 | 1.65 | 1.64 | 1.65 | 1.01 |
| Trp | TGG | 1.00 | 1.00 | 1.00 | 1.00 | 1.00 | 1.00 | 1.00 | 1.00 |
| Tyr | TAC | 0.66 | 0.41 | 0.84 | 0.84 | 0.41 | 0.39 | 0.41 | 0.63 |
| Tyr | TAT | 1.34 | 1.59 | 1.16 | 1.16 | 1.59 | 1.61 | 1.59 | 1.37 |
| Val | GTA | 0.77 | 1.42 | 1.16 | 1.16 | 1.45 | 1.43 | 1.42 | 0.85 |
| Val | GTC | 0.92 | 0.50 | 0.82 | 0.81 | 0.50 | 0.51 | 0.52 | 0.85 |
| Val | GTG | 0.56 | 0.64 | 0.89 | 0.90 | 0.64 | 0.64 | 0.65 | 0.58 |
| Val | GTT | 1.75 | 1.43 | 1.14 | 1.14 | 1.42 | 1.42 | 1.41 | 1.73 |

* B. men: *B. mentosum*, B. epi: B. *epiphytum*, B: plu: B. *plumosum*, B. wed: *B. weddellii*, B. exa: *B. exaltatum*, B. gra: *B. granulosum*, B. reg: *B. regnellii*, B. ste: *B. steyermarkii*

**Supplementary Table 6.** Distribution and frequency of the repeat sequences in *Bulbophyllum* plastid genomes.

| Species | Direction | | | Total general |
|---|---|---|---|---|
| | Forward | Palindromic | Reverse | |
| *B. mentosum* | 6 | 17 | 2 | 25 |
| *B. epiphytum* | 12 | 20 | 1 | 33 |
| *B. plumosum* | 10 | 20 | 1 | 31 |
| *B. weddellii* | 10 | 18 | 2 | 30 |
| *B. exaltatum* | 9 | 14 | 4 | 27 |
| *B. granulosum* | 11 | 20 | 3 | 34 |
| *B. regnellii* | 11 | 21 | 1 | 33 |
| *B. steyermarkii* | 9 | 23 | 4 | 36 |
| Total | 78 | 153 | 18 | 249 |

**Supplementary Table 7.** Frequency of repeat sequence length for each *Bulbophyllum* species.

| Length | *B. men* | *B. epi* | *B. plu* | *B. wed* | *B. exa* | *B. gra* | *B. reg* | *B. ste* | Total |
|---|---|---|---|---|---|---|---|---|---|
| 30 | 12 | 12 | 14 | 10 | 9 | 10 | 13 | 12 | 92 |
| 31 | 1 | 1 | 1 | 2 | 2 | 2 | 1 | 3 | 13 |
| 32 | 2 | 10 | 3 | 4 | 3 | 8 | 3 | 2 | 35 |
| 33 | 1 | 0 | 0 | 2 | 1 | 0 | 4 | 2 | 10 |
| 34 | 1 | 1 | 3 | 2 | 2 | 1 | 2 | 0 | 12 |
| 35 | 0 | 0 | 0 | 0 | 0 | 0 | 1 | 1 | 2 |
| 36 | 1 | 2 | 2 | 2 | 2 | 1 | 1 | 2 | 13 |
| 37 | 1 | 1 | 2 | 1 | 1 | 2 | 1 | 3 | 12 |
| 38 | 0 | 1 | 0 | 0 | 0 | 1 | 0 | 0 | 2 |
| 39 | 3 | 2 | 3 | 5 | 4 | 3 | 3 | 3 | 26 |
| 40 | 1 | 0 | 0 | 0 | 0 | 1 | 0 | 0 | 2 |
| 41 | 0 | 0 | 0 | 0 | 0 | 1 | 0 | 0 | 1 |
| 43 | 0 | 0 | 0 | 0 | 0 | 1 | 0 | 0 | 1 |
| 44 | 0 | 0 | 0 | 0 | 0 | 0 | 0 | 4 | 4 |
| 46 | 1 | 1 | 1 | 1 | 1 | 1 | 1 | 1 | 8 |
| 47 | 0 | 1 | 1 | 0 | 2 | 1 | 1 | 1 | 7 |
| 48 | 0 | 0 | 0 | 0 | 0 | 0 | 0 | 1 | 1 |
| 50 | 0 | 1 | 0 | 0 | 0 | 0 | 0 | 0 | 1 |
| 52 | 0 | 0 | 0 | 0 | 0 | 0 | 1 | 1 | 2 |
| 53 | 1 | 0 | 0 | 0 | 0 | 0 | 0 | 0 | 1 |
| 54 | 0 | 0 | 0 | 0 | 0 | 1 | 0 | 0 | 1 |
| 59 | 0 | 0 | 1 | 1 | 0 | 0 | 1 | 0 | 3 |

* B. men: *B. mentosum,* B. epi: B. *epiphytum,* B: plu: B. *plumosum,* B. wed: *B. weddellii*, B. exa: *B. exaltatum*, B. gra: *B. granulosum,* B. reg: *B. regnellii*, B. ste: *B. steyermarkii.*

**Supplementary Table 8.** Distribution and frequency of the simple single sequence repeats (SSRs) among *Bulbophyllum* genomes.

| Specie | Total | Unit size | | | | | | Total per region | | | Total per region | | | A/T SSRs | % A/T SSRs |
|---|---|---|---|---|---|---|---|---|---|---|---|---|---|---|---|
| | | Mono | Di | Tri | Tetra | Penta | Hexa | CDS | IGS | INTRON | IR | LSC | SSC | | |
| *B. mentosum* | 80 | 57 | 10 | 3 | 5 | 5 | 0 | 14 | 53 | 13 | 6 | 63 | 11 | 74 | 92.50 |
| *B. epiphytum* | 80 | 61 | 9 | 0 | 6 | 1 | 3 | 15 | 54 | 11 | 8 | 60 | 12 | 73 | 91.25 |
| *B. plumosum* | 72 | 47 | 14 | 0 | 7 | 3 | 1 | 14 | 45 | 13 | 6 | 58 | 8 | 67 | 93.06 |
| *B. weddellii* | 61 | 48 | 8 | 0 | 5 | 0 | 0 | 10 | 42 | 9 | 4 | 49 | 8 | 57 | 93.44 |
| *B. exaltatum* | 56 | 42 | 8 | 0 | 5 | 1 | 0 | 9 | 38 | 9 | 4 | 46 | 6 | 52 | 92.86 |
| *B. granulosum* | 59 | 37 | 13 | 2 | 6 | 1 | 0 | 13 | 35 | 11 | 4 | 44 | 11 | 51 | 86.44 |
| *B. regnellii* | 56 | 31 | 11 | 6 | 7 | 1 | 0 | 13 | 36 | 7 | 4 | 40 | 12 | 46 | 82.14 |
| *B. steyermarkii* | 63 | 42 | 11 | 1 | 6 | 2 | 1 | 11 | 41 | 11 | 7 | 51 | 5 | 54 | 85.71 |

*CDS: gene encoding proteins, IGS: intergenic spacer, LSC: larger single copy region, SSC: small single copy region, IR: invert region.

**Supplementary Table 9.** Distribution of polymorphic SSRs and their specific primer pairs that are present in at least four species.

| Primer | Position | Region | Location | SSR Type | Primer sequence (5′-3′) | Tm | Length |
|---|---|---|---|---|---|---|---|
| Bulbo ssr1 | *psbA-matK* | LSC | IGS | (A) 11, (T)17 | CCATAGGAATRACCAAAACT | 54.1 | 240 |
| | | | | | TATACATAGGGAAAGTCGTGTG | 54.5 | |
| Bulbo ssr2 | *trnK-matK* | LSC | IGS | (A) 13 | GCTTGCARTTTTCATTGCAC | 59.5 | 267 |
| | | | | | CGGATTACATAGAGAACGTATTTGG | 60.2 | |
| Bulbo ssr3 | *matK-trnK* | LSC | IGS | (AAAG) 4 | GAGACAGAGAGCCCAATCTA | 54.4 | 232 |
| | | | | | KAGACRGATGTGTAGAAGAAAT | 5.1 | |
| Bulbo ssr4 | *matK-trnK* | LSC | IGS | (A) 12 | TCTTCTACACATCYGTCTMCAA | 59.7 | 181 |
| | | | | | GCAACGAGCTTCCTTTCTTAAT | 59.1 | |
| Bulbo ssr5 | *rps16* | LSC | INTRON | (AT) 5, (TA) 6 | TAGATGGCTCATTGGGATA | 54 | 226 |
| | | | | | ACTTGAGTTAGGAGTACGAATG | 53 | |
| Bulbo ssr6 | *rps16* | LSC | INTRON | (T) 10 | AGGCAGCAACATACCTTT | 53.2 | 218 |
| | | | | | GTKACAATCAATACGTTAGACC | 53.9 | |
| Bulbo ssr7 | *rps16-trnQ* | LSC | IGS | (A) 14 | GCTTTCTACCACATCGTTT | 52.8 | 299 |
| | | | | | GKATCTYTTATGATGGACAA | 53.4 | |
| Bulbo ssr8 | *rps16-trnQ* | LSC | IGS | (A) 11, (A) 12 | GGAATTGTCCATCATAARAG | 52 | 180 |
| | | | | | GTCCCAGATCGTTTGYATM | 52.7 | |
| Bulbo ssr9 | *psbK-psbI* | LSC | IGS | (A) 12 | GTTATGCCGRTTATACCTTTAC | 54.8 | 194 |
| | | | | | ATCGAGGGTTYCTAATGTCYA | 55.4 | |
| Bulbo ssr10 | *psbK-psbI* | LSC | IGS | (A) 12 | CWTTTGAATAARGGAAGGG | 54.7 | 295 |
| | | | | | CACCTATTTTACGACACACACT | 54.5 | |
| Bulbo ssr11 | *psbK-psbI* | LSC | IGS | (A) 10 | GTGTGTGTCGTAAAATAGGTG | 53.2 | 129 |
| | | | | | CGAAGATGAAGAGAGAAACA | 53.1 | |
| Bulbo ssr12 | *trnG-trnR* | LSC | IGS | (T) 12 | CGACTATAACCCCTAGCCTTCC | 60.3 | 183 |
| | | | | | GACGCCCTTCATTCCTATTTTC | 61.1 | |

**Supplementary Table 9.** Cont.

| Primer | Position | Region | Location | SSR Type | Primer sequence (5′-3′) | Tm | Length |
|---|---|---|---|---|---|---|---|
| Bulbo ssr13 | *trnR-atpA* | LSC | IGS | (A)15 | TAATGGATAGGACAKAGGTCTT | 54.6 | 299 |
| | | | | | AAAYCCTTTTGAGAGAAGC | 53.7 | |
| Bulbo ssr14 | *atpF* | LSC | INTRON | (A) 15 | GACTCTTCAGACCAGACAAAA | 54.5 | 299 |
| | | | | | CATTAACTGGAAAGATGGGT | 54.2 | |
| Bulbo ssr15 | *atpI-rps2* | LSC | IGS | (A) 11 | CGGATACCGAKAAATCACA | 57 | 275 |
| | | | | | GGCCAACGATGATGCTAT | 57 | |
| Bulbo ssr16 | *rps2-rpoC2* | LSC | IGS | (T) 12 | ATGACCAAAATGAACTCCTGCT | 60 | 317 |
| | | | | | TCCATGAGACATCAGACCAATC | 59.9 | |
| Bulbo ssr17 | *rpoC2* | LSC | CDS | (T) 11 | AGATYCTGAGAGAAAAGTGTTG | 52.3 | 300 |
| | | | | | AAGAAGTCGAGTAGGTGGATTA | 54.9 | |
| Bulbo ssr18 | *rpoB-trnC* | LSC | IGS | (AAGT)3, (AAAT) 4 | CGGGCTCRATATCTTATCTACGTAT | 58 | 380 |
| | | | | | ATCTCAAAACACGCRTCGTT | 56.4 | |
| Bulbo ssr19 | *rpoB-trnC* | LSC | IGS | (ATTT) 3 | AACGAYGCGTGTTTTGAGAT | 58.2 | 400 |
| | | | | | GGATTCGTSAAGTTCGATCA | 58.6 | |
| Bulbo ssr20 | *petN-psbM* | LSC | IGS | (TATAT) 4 | CTCTTTCACTTGTAGTATGGG | 53.4 | 376 |
| | | | | | CCAACTGCTTKAATTCCTGA | 53.5 | |
| Bulbo ssr21 | *trnS-psbZ* | LSC | IGS | (A) 10 | ACTCAGCCATCTCTCCCAAATA | 60.1 | 314 |
| | | | | | CCAACCATCAGAAGAAGCAAAT | 60.5 | |
| Bulbo ssr22 | *psaA-ycf3* | LSC | IGS | (T) 14 | CRAAGTGCCCATAGACTTTAAT | 57.5 | 261 |
| | | | | | AGGCGTTTTGAATAAGACCA | 57.3 | |
| Bulbo ssr23 | *ycf3-2* | LSC | INTRON | (T) 13 | TTCACCAAGCGTAGGTTTCTTT | 60.2 | 170 |
| | | | | | AATTCCGACAACTTCAGGAGAA | 60.1 | |
| Bulbo ssr24 | *trnS-rps4* | LSC | IGS | (A) 11 | CTCTCTCCGTTTMTTTTMAT | 53.7 | 278 |
| | | | | | ATGAGTTGTTGGTYGTAGAA | 53.1 | |

**Supplementary Table 9.** Cont.

| Primer | Position | Region | Location | SSR Type | Primer sequence (5′-3′) | Tm | Length |
|---|---|---|---|---|---|---|---|
| Bulbo ssr25 | *atpB-rbcL* | LSC | IGS | (T) 12 | CCACYCAATCGAATCCAA | 60.3 | 232 |
| | | | | | AGAAAGAATAGAGGACCACCCC | 59.8 | |
| Bulbo ssr26 | *accD-psaI* | LSC | IGS | (A) 12 | TTMARCTACATGGTTTCCTTCC | 60.5 | 297 |
| | | | | | TCRATAKAGGGATSCTTATTCC | 59.6 | |
| Bulbo ssr27 | *petA-psbJ* | LSC | IGS | (A) 11 | TTTGTGGGATGTCTGAAACT | 55.5 | 133 |
| | | | | | AGACGATTAPGAAGGCTAAKAA | 56.5 | |
| Bulbo ssr28 | *petA-psbJ* | LSC | IGS | (A) 12 | ATTGTGTCGTCTTGTATCGAAAG | 59 | 300 |
| | | | | | TKAAACATCCTGCAAATMCT | 58.2 | |
| Bulbo ssr29 | *trnP-psaJ* | LSC | IGS | (T) 13 | ATGAATCTCTCGTAACTKBCY | 53.4 | 269 |
| | | | | | TCTGMTGGGTCATTATACAT | 53.1 | |
| Bulbo ssr30 | *psaJ-rpl33* | LSC | IGS | (A) 13 | GTAACACGGCTCCAAWTTTGC | 58.7 | 143 |
| | | | | | RATTTMCCCTAKAATGGATCCACA | 58.4 | |
| Bulbo ssr31 | *psaJ-rpl33* | LSC | IGS | (A) 14, (TAT) 4 | AAATGTGGATCCATTMTAGGGK | 57.5 | 396 |
| | | | | | GCCATGAACCTCCTTTTCTTTT | 57.4 | |
| Bulbo ssr32 | *clpP1* | LSC | INTRON | (A) 12, (T) 10 | GTGACGCTKAAATTGACTCTTG | 59.2 | 262 |
| | | | | | TACCAAACGTCTAGCATTCCCT | 60 | |
| Bulbo ssr33 | *clpP2* | LSC | INTRON | (T) 13 | TCTCGATGAAGTCGGTTGATTA | 59.7 | 286 |
| | | | | | GCCCATTMAGGAACAAGAAA | 60.1 | |
| Bulbo ssr34 | *clpP2* | LSC | INTRON | (T) 12 | TTAGGTTCTTGTTCTACTCCG | 54.7 | 127 |
| | | | | | CATCGAATATTTTGGGGAAAGG | 55.5 | |
| Bulbo ssr35 | *clpP-psbB* | LSC | IGS | (TA) 8, (T) 10, (TAT) 4 | CAAGTCGCACTATACGTCAACC | 59.7 | 379 |
| | | | | | TCCTCTATGAACCTTCCAGTCC | 59.6 | |
| Bulbo ssr36 | *psbB-psbT* | LSC | IGS | (T) 13 | TTTGYCATCTTTTGCCTCTC | 60.3 | 496 |
| | | | | | CACACCTATTYGTTTTGGATCA | 59.9 | |

**Supplementary Table 9.** Cont.

| Primer | Position | Region | Location | SSR Type | Primer sequence (5′-3′) | Tm | Length |
|---|---|---|---|---|---|---|---|
| Bulbo ssr37 | *petB* | LSC | INTRON | (A) 13 | CATAGGGTCTCAACAAGAGAATCC | 60.4 | 259 |
| | | | | | TCAAGCCGAAAMACACAAATAC | 60 | |
| Bulbo ssr38 | *petD* | LSC | INTRON | (T) 14 | GGCTCCGTAAGATCCCTAGAAT | 60 | 236 |
| | | | | | CCCKGTTCTTCCTTAGATCCCT | 60 | |
| Bulbo ssr39 | *rpoA* | LSC | CDS | (T) 10 | GCAAAATGTTTCTGTARAGTGYC | 60.2 | 295 |
| | | | | | ATGCAGAGGAAGAGGACATGAA | 61.2 | |
| Bulbo ssr40 | *rps8-rpl14* | LSC | IGS | (T) 12 | ATGTCCYTACCCATGACGAAC | 58.8 | 265 |
| | | | | | AGTTTCATTAGCACCCGAAGTA | 58 | |
| Bulbo ssr41 | *rpl16* | LSC | INTRON | (T) 11 | CAATGGAGCTCTCAACAAAATMTG | 58.6 | 374 |
| | | | | | AGGCAGTGTTATAAAGCATCAACA | 59 | |
| Bulbo ssr42 | *rpl16* | LSC | INTRON | (T) 11 | TCTCTMTTTCTATCATCYTTCC | 54.4 | 286 |
| | | | | | TCAGTGTGTGACTCGTTAGTTT | 55.1 | |
| Bulbo ssr43 | *rps12-trnV* | IR | IGS | (T) 14 | TCTGGGCTCTTCTATCTTCTAC | 54.7 | 161 |
| | | | | | CTACAGGATCAACAAACCTATG | 54.1 | |
| Bulbo ssr44 | *rps12-trnV* | IR | IGS | (TATTA) 3 | GAGATCCTTTCGATGACCTAT | 54.9 | 335 |
| | | | | | AGAGACAAAATGTAGGACTGGT | 54.6 | |
| Bulbo ssr45 | *ndhD* | SSC | CDS | (T) 11 | CAAGATCAAGGYACAAATTC | 55.8 | 380 |
| | | | | | TTATCTHYCTGGTTGGTTATAG | 55.1 | |
| Bulbo ssr46 | *ycf1* | SSC | CDS | (ATTTTC) 3 | MTWCMATTYCTKAAAATCCY | 55.3 | 282 |
| | | | | | ATGRGCAGAGAAMAAGAAT | 54.7 | |

*CDS: gene encoding proteins, IGS: intergenic spacer, LSC: larger single copy region, SSC: small single copy region, IR: invert region, TM: Primer Melting Temperature.

**Supplementary Table 10**. Sequence variability (SV), AT and GC content, and length of 148 sequences (CDS, IGS and introns) that are flanked by the same exon and longer than 150 pairs. The number of mutations and indels events were counted using a *B. steyermarkii* plastid genome as reference.

| Sequence | SV | AT % | GC % | Length | *Sequence* | SV | AT % | GC % | Length |
|---|---|---|---|---|---|---|---|---|---|
| *trnR-atpA* | 19.58 | 81.3 | 18.7 | 186 | *rpl22-rps19* | 4.23 | 76 | 24 | 263 |
| *trnM-aptE* | 17.44 | 73.8 | 26.2 | 187 | *atpI-rps2* | 4.17 | 78.4 | 21.6 | 304 |
| *ccsA-ndhD* | 17.16 | 77.2 | 22.8 | 297 | *rpl23-trnI* | 3.97 | 66.5 | 33.5 | 172 |
| *clpP-psbB* | 15.14 | 80.5 | 19.5 | 847 | *trnR-trnN* | 3.93 | 58.7 | 41.3 | 563 |
| *trnS-trnG* | 14.79 | 81.4 | 18.6 | 1075 | *ccsA* | 3.93 | 62.5 | 37.5 | 978 |
| *psbB-psbT* | 14.67 | 86.5 | 13.5 | 692 | *rpl22* | 3.81 | 68.4 | 31.6 | 375 |
| *atpH-atpI* | 12.12 | 72.4 | 27.6 | 782 | *rps16* | 3.78 | 65.8 | 34.2 | 288 |
| *psbK-psbI* | 11.95 | 76.1 | 23.9 | 507 | *rrn4.5s-rrn5s* | 3.65 | 55.8 | 44.2 | 221 |
| *rpl32-trnL* | 11.93 | 77.8 | 22.2 | 676 | *rpoC1 intron* | 3.64 | 63.9 | 36.1 | 771 |
| *matK-trnK* | 11.76 | 77.9 | 22.1 | 1277 | *trnV (UAC) intron* | 3.46 | 62.3 | 37.7 | 583 |
| *psbI-trnS* | 11.35 | 80.4 | 19.6 | 203 | *infA* | 3.42 | 63.5 | 36.5 | 234 |
| *trnE-trnT* | 10.89 | 80.5 | 19.5 | 1140 | *clpP* | 3.19 | 59.1 | 40.9 | 615 |
| *trnW-trnP* | 10.67 | 69.3 | 30.7 | 168 | *ycf3 intron* | 3.19 | 67.5 | 32.5 | 756 |
| *psaJ-rpl33* | 10.48 | 79.5 | 20.5 | 704 | *petB intron* | 3.18 | 66.5 | 33.5 | 728 |
| *rps18-rpl20* | 10.33 | 72 | 28 | 272 | *rps12-trnV* | 3.09 | 63.7 | 36.3 | 2058 |
| *rpl16 intron* | 10.32 | 75.5 | 24.5 | 1380 | *rpoA* | 3.05 | 65.2 | 34.8 | 1029 |
| *petA-psbJ* | 10.16 | 72 | 28 | 1011 | *rps3* | 3.03 | 66.4 | 33.6 | 657 |
| *trnP-psaJ* | 10.06 | 76.7 | 23.3 | 378 | *rps8* | 3.02 | 67.9 | 32.1 | 396 |
| *petN-psbM* | 10.04 | 79.2 | 20.8 | 883 | *rpl33* | 2.99 | 65 | 35 | 201 |
| *accD-psaI* | 9.93 | 75 | 25 | 911 | *cemA* | 2.75 | 66.5 | 33.5 | 690 |
| *ycf1* | 9.89 | 72.4 | 27.6 | 5484 | *trnI-ndhB* | 2.75 | 62.7 | 37.3 | 550 |
| *rpoB-trnC* | 9.84 | 73.6 | 26.4 | 1553 | *atpF* | 2.70 | 63.9 | 36.1 | 555 |
| *trnK-rps16* | 9.73 | 76.6 | 23.4 | 603 | *rpl16* | 2.70 | 56.7 | 43.3 | 408 |
| *ndhC* | 9.73 | 63.6 | 36.4 | 375 | *rps18* | 2.68 | 64 | 36 | 297 |
| *trnV-trnM* | 9.71 | 68.7 | 31.3 | 175 | *trnA-rrn23s* | 2.63 | 58.7 | 41.3 | 157 |
| *rps8-rpl14* | 9.71 | 81.1 | 18.9 | 225 | *rpoC2* | 2.62 | 63.7 | 36.3 | 4170 |
| *clpP intron 2* | 9.50 | 75.5 | 24.5 | 1111 | *trnN-ycf1* | 2.47 | 62.5 | 37.5 | 329 |
| *rps16-trnQ* | 9.27 | 86.3 | 13.7 | 1079 | *atpE* | 2.44 | 59.8 | 40.2 | 411 |
| *rps11-rpl36* | 9.09 | 76.7 | 23.3 | 159 | *rpl32* | 2.30 | 69.3 | 30.7 | 174 |
| *ndhC-trnV* | 8.96 | 74.1 | 25.9 | 323 | *rps2* | 2.25 | 62.5 | 37.5 | 711 |
| *cemA-petA* | 8.81 | 71.8 | 28.2 | 226 | *rpl14* | 2.17 | 60.9 | 39.1 | 369 |
| *trnC-petN* | 8.72 | 69.6 | 30.4 | 838 | *ycf4* | 2.16 | 60.3 | 39.7 | 555 |
| *petD-rpoA* | 8.59 | 70.4 | 29.6 | 175 | *ndhB* | 2.11 | 63.5 | 36.5 | 1542 |
| *trnT-psbD* | 8.38 | 69.4 | 30.6 | 947 | *rpoC1* | 2.04 | 61.4 | 38.6 | 2067 |
| *trnD-trnY* | 8.30 | 70.8 | 29.2 | 270 | *petB* | 2.00 | 60.6 | 39.4 | 648 |
| *ycf3-trnS* | 8.29 | 68.7 | 31.3 | 216 | *petA* | 1.87 | 61.7 | 38.3 | 963 |
| *trnS-rps4* | 8.26 | 73.3 | 26.7 | 323 | *petD* | 1.86 | 62.2 | 37.8 | 492 |

**Supplementary Table 10.** Cont.

| Sequence | SV | AT % | GC % | Length | *Sequence* | SV | AT % | GC % | Length |
|---|---|---|---|---|---|---|---|---|---|
| *rpl33-rps18* | 8.06 | 73 | 27 | 189 | *ndhB-rps7* | 1.86 | 64.9 | 35.1 | 323 |
| *rpl16-rps3* | 8.05 | 83.3 | 16.7 | 212 | *rpoB* | 1.84 | 62.1 | 37.9 | 3213 |
| *trnmF-rps14* | 7.93 | 64.3 | 35.7 | 164 | *psbA* | 1.79 | 58.3 | 41.7 | 1062 |
| *petL-petG* | 7.89 | 70.4 | 29.6 | 189 | *rrn5s-trnR* | 1.71 | 58.6 | 41.4 | 234 |
| *rps16 intron* | 7.83 | 70.5 | 29.5 | 912 | *atpA* | 1.71 | 60.6 | 39.4 | 1524 |
| *psaA-ycf3* | 7.78 | 73.3 | 26.7 | 591 | *rps11* | 1.68 | 57.1 | 42.9 | 417 |
| *ycf4-cemA* | 7.64 | 74.8 | 25.2 | 750 | *rps4* | 1.65 | 61.3 | 38.7 | 606 |
| *psbE-petL* | 7.50 | 74.6 | 25.4 | 1222 | *ycf2* | 1.62 | 62.2 | 37.8 | 6909 |
| *rps15-ycf1* | 7.41 | 78.1 | 21.9 | 467 | *ndhB intron* | 1.48 | 61.4 | 38.6 | 699 |
| *trnG-trnfM* | 7.30 | 68.1 | 31.9 | 177 | *rbcL* | 1.37 | 56.4 | 43.6 | 1470 |
| *ndhD* | 7.29 | 73 | 27 | 1577 | *psbH* | 1.35 | 62.7 | 37.3 | 222 |
| *psbZ-trnG* | 7.28 | 70.4 | 29.6 | 226 | *atpB* | 1.34 | 57.2 | 42.8 | 1497 |
| *psaI-ycf4* | 7.11 | 70.3 | 29.7 | 434 | *psbB* | 1.31 | 56.2 | 43.8 | 1527 |
| *trnK-matK* | 6.88 | 74.1 | 25.9 | 278 | *psbC* | 1.27 | 55.9 | 44.1 | 1422 |
| *psbA-trnK* | 6.81 | 76.7 | 23.3 | 277 | *trnA (UGC) intron* | 1.25 | 50.1 | 49.9 | 815 |
| *rbcL-accD* | 6.58 | 70.5 | 29.5 | 730 | *psaA* | 1.24 | 57.1 | 42.9 | 2253 |
| *atpF intron* | 6.45 | 70.7 | 29.3 | 937 | *psbE* | 1.19 | 61.2 | 38.8 | 252 |
| *rps4-trnT* | 6.34 | 72.8 | 27.2 | 342 | *ycf2-trnL* | 1.16 | 66.8 | 33.2 | 977 |
| *rpl20-rps12* | 6.34 | 67.2 | 32.8 | 766 | *psbK* | 1.11 | 65.7 | 34.3 | 180 |
| *clpP intron* | 6.28 | 70.1 | 29.9 | 697 | *rps12 intron* | 1.10 | 60.6 | 39.4 | 547 |
| *atpB-rbcL* | 6.20 | 80.1 | 19.9 | 1133 | *rps19* | 1.08 | 60.8 | 39.2 | 279 |
| *rps15* | 6.18 | 69.2 | 30.8 | 273 | *rrn16-trnI* | 1.00 | 52.1 | 47.9 | 309 |
| *rps12-clpP* | 6.11 | 70.6 | 29.4 | 154 | *ycf3* | 0.99 | 62.7 | 37.3 | 507 |
| *petB-petD* | 5.95 | 71.8 | 28.2 | 185 | *trnI (GAU) intron* | 0.96 | 51 | 49 | 952 |
| *trnQ-psbK* | 5.92 | 75.2 | 24.8 | 379 | *psaB* | 0.95 | 58.9 | 41.1 | 2205 |
| *rpoC2-rpoC1* | 5.88 | 63.5 | 36.5 | 187 | *psbD* | 0.94 | 58.2 | 41.8 | 1062 |
| *accD* | 5.87 | 68 | 32 | 1482 | *atpI* | 0.94 | 63.4 | 36.6 | 744 |
| *psbH-petB* | 5.65 | 73.6 | 26.4 | 184 | *trnV-rrn16s* | 0.87 | 53.1 | 46.9 | 229 |
| *petD intron* | 5.58 | 67.2 | 32.8 | 812 | *psaC* | 0.81 | 59.1 | 40.9 | 246 |
| *rps2-rpoC2* | 5.58 | 74.3 | 25.7 | 268 | *rpl23* | 0.74 | 61.9 | 38.1 | 270 |
| *matK* | 4.68 | 68.6 | 31.4 | 1536 | *rps14* | 0.66 | 59.5 | 40.5 | 303 |
| *ycf3 intron 2* | 4.58 | 65.4 | 34.6 | 734 | *rps12* | 0.54 | 57.9 | 42.1 | 372 |
| *trnS-psbZ* | 4.52 | 69.5 | 30.5 | 215 | *psbZ* | 0.53 | 66.3 | 33.7 | 189 |
| *trnL-trnF* | 4.49 | 72.9 | 27.1 | 254 | *rps7* | 0.43 | 58.8 | 41.2 | 468 |
| *rpl20* | 4.42 | 64.7 | 35.3 | 387 | *atpH* | 0.41 | 56.6 | 43.4 | 246 |
| *psbM-trnD* | 4.35 | 76.2 | 23.8 | 1113 | *rpl2 intron* | 0.30 | 59.3 | 40.7 | 664 |
| *trnG (UCC) intron* | 4.30 | 68.1 | 31.9 | 683 | *rpl2* | 0.24 | 55.6 | 44.4 | 822 |

**Supplementary Table 11.** Specific primer pairs for the ten most-variable plastid regions in Neotropical *Bulbophyllum*.

| Nro. | Hypervariable sequence | Region | Location | Primer sequence (5′-3′) | Length | Tm | Product |
|---|---|---|---|---|---|---|---|
| 1 | *trnR-atpA* | LSC | IGS | CCTTTGGTATAGGTTCAAATCC | 22 | 56.7 | 261 |
| | | | | CCAAGATATTTACCGAAGAAGC | 22 | 57.1 | |
| 2 | *trnM-atpE* | LSC | IGS | AGAGTATTGCTTTCATACGG | 20 | 52.2 | 226 |
| | | | | GCTGTCAAAGTKATTTCTTC | 20 | 52.5 | |
| 3 | *ccsA-ndhD* | SSC | IGS | GGGATCAATCTATTAGGAATCG | 22 | 56.7 | 984 |
| | | | | TTATCTHYCTGGTTGGTTATAG | 22 | 55.1 | |
| 4 | *clpP-psbB* | LSC | IGS | ACAAGTCGCACTATACGTCAA | 21 | 56 | 870 |
| | | | | CAACAGTATGAACACGATACCA | 22 | 56.1 | |
| 5 | *trnS-trnG* | LSC | IGS | CTTTAATCCACTCAGCCATC | 20 | 54.9 | 757 |
| | | | | TGTCCAAYCAAAGTAATCAK | 20 | 53.6 | |
| 6 | *psbB-psbT* | LSC | IGS | TCCAACTACAAGGAGACAA | 19 | 51.3 | 631 |
| | | | | GTCGAAACTAAGAGGAATGT | 20 | 51 | |
| 7 | *atpH-atpI* | LSC | IGS | GGATTCATGGTAAGTTCCTY | 20 | 53.6 | 738 |
| | | | | GGATTCACCTATATAAGCCG | 20 | 54 | |
| 8 | *rpl23-trnL* | SSC | IGS | TCTTTAGAGGCAGTAAAAGC | 20 | 51.9 | 764 |
| | | | | GTGTCTACCAATTTCACCAT | 20 | 52.3 | |
| 9 | *psbk-psbI* | LSC | IGS | TCTCTTAGCCTTTGTTTGG | 19 | 53.2 | 571 |
| | | | | CGAAGATGAAGAGAGAAACA | 20 | 53 | |
| 10 | *matK-trnK* | LSC | IGS | GATATARGAAGTTTTGTTSCCG | 22 | 55.5 | 1,050 |
| | | | | GGGTCTAGTKAATAAATGGATAGAG | 25 | 56.3 | |

*LSC: large single copy region, SSC: small single copy region, IGS: intergenic spacer, TM: primer melting temperature.

**Supplementary Figure 1.** Organization of *Bulbophyllum* plastid genomes. A) *B. exaltatum*, B) *B. weddellii*, C) *B. steyermarkii*, D) *B. epiphytum*, E) *B. plumosum*, F) *B. mentosum* and G) *B. granulosum.*

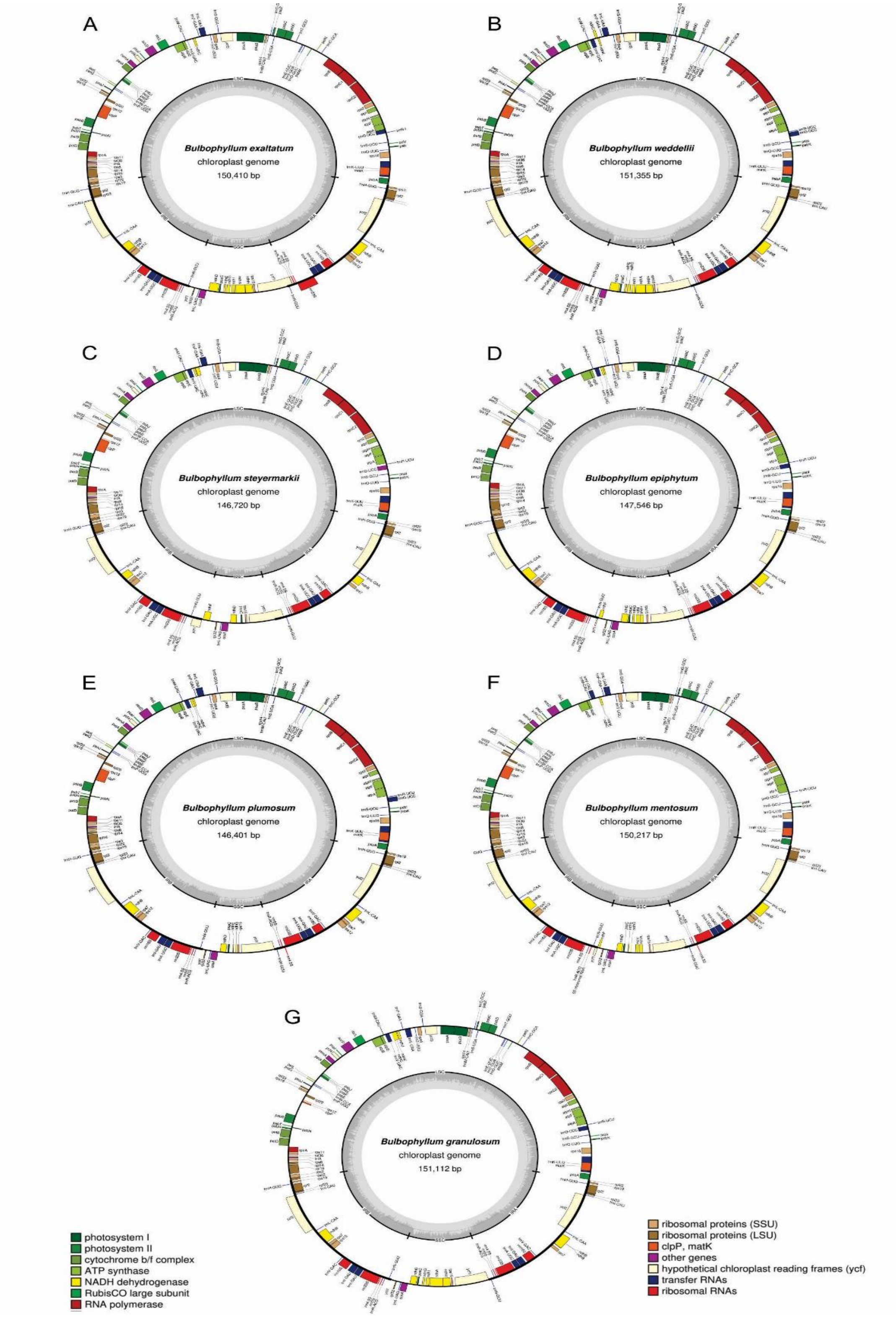

**Supplementary Figure 2.** Comparison among the eight plastid genomes. The figure represents the total length of plastomes and length of LSC, SSC, and IR region.

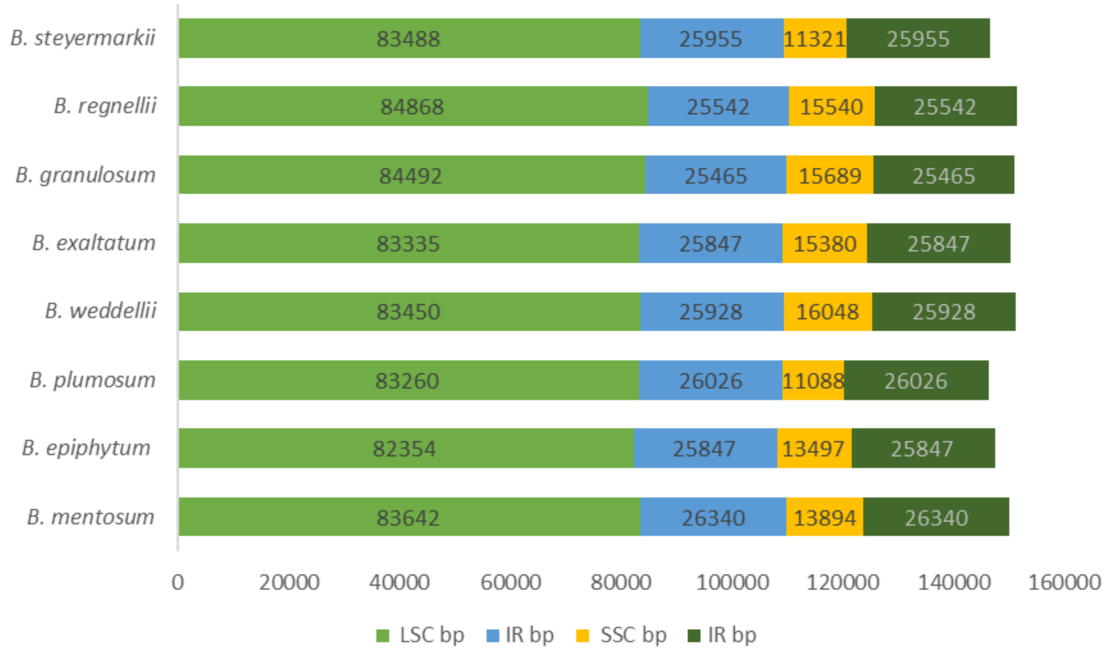


*LSC: large single copy region, SSC: small single copy region, IR: invert region

**Supplementary Figure 3.** Adenine-thymine content expressed as a percentage of coding and non-coding regions.

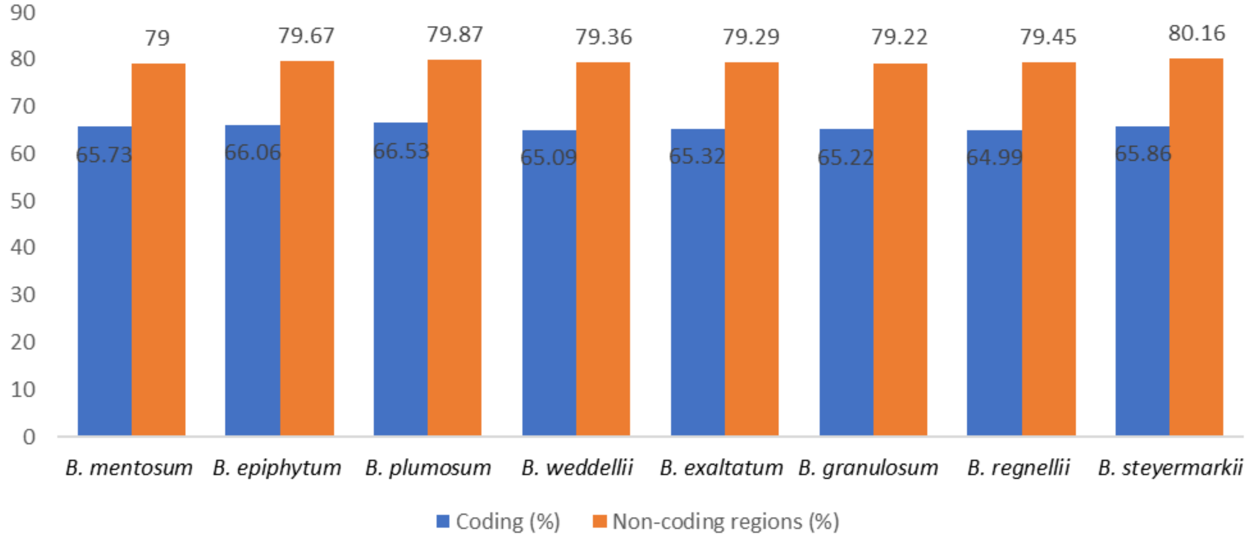

**Supplementary Figure 4.** Sequence variability (SV) and parsimony informative sites (PIS) of nrITS and the top ten most-variable regions.

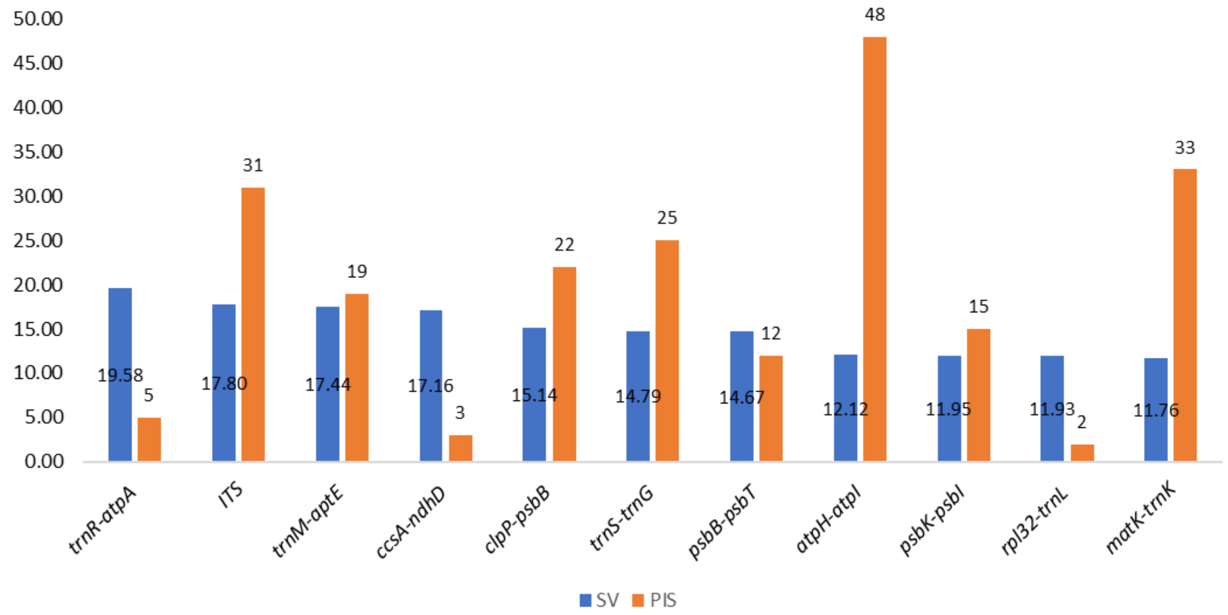

Supplement: Supplementary file 1 [file Data_Sheet_1.PDF]
